# Genetic Alterations of TRAF Proteins in Human Cancers

**DOI:** 10.3389/fimmu.2018.02111

**Published:** 2018-09-20

**Authors:** Sining Zhu, Juan Jin, Samantha Gokhale, Angeli M. Lu, Haiyan Shan, Jianjun Feng, Ping Xie

**Affiliations:** ^1^Department of Cell Biology and Neuroscience, Rutgers University, Piscataway, NJ, United States; ^2^Graduate Program in Cellular and Molecular Pharmacology, Rutgers University, Piscataway, NJ, United States; ^3^Department of Pharmacology, Anhui Medical University, Hefei, China; ^4^Department of Obstetrics and Gynecology, The Affiliated Suzhou Hospital of Nanjing Medical University, Suzhou, China; ^5^Engineering Research Center of the Modern Technology for Eel Industry, Ministry of Education of the People's Republic of China, Fisheries College of Jimei University, Xiamen, China; ^6^Member, Rutgers Cancer Institute of New Jersey, New Brunswick, NJ, United States

**Keywords:** TRAFs, cancer, oncogenes, tumor suppressor genes, NF-κB, MAPK

## Abstract

The tumor necrosis factor receptor (TNF-R)-associated factor (TRAF) family of cytoplasmic adaptor proteins regulate the signal transduction pathways of a variety of receptors, including the TNF-R superfamily, Toll-like receptors (TLRs), NOD-like receptors (NLRs), RIG-I-like receptors (RLRs), and cytokine receptors. TRAF-dependent signaling pathways participate in a diverse array of important cellular processes, including the survival, proliferation, differentiation, and activation of different cell types. Many of these TRAF-dependent signaling pathways have been implicated in cancer pathogenesis. Here we analyze the current evidence of genetic alterations of *TRAF* molecules available from The Cancer Genome Atlas (TCGA) and the Catalog of Somatic Mutations in Cancer (COSMIC) as well as the published literature, including copy number variations and mutation landscape of TRAFs in various human cancers. Such analyses reveal that both gain- and loss-of-function genetic alterations of different TRAF proteins are commonly present in a number of human cancers. These include pancreatic cancer, meningioma, breast cancer, prostate cancer, lung cancer, liver cancer, head and neck cancer, stomach cancer, colon cancer, bladder cancer, uterine cancer, melanoma, sarcoma, and B cell malignancies, among others. Furthermore, we summarize the key *in vivo* and *in vitro* evidence that demonstrates the causal roles of genetic alterations of TRAF proteins in tumorigenesis within different cell types and organs. Taken together, the information presented in this review provides a rationale for the development of therapeutic strategies to manipulate TRAF proteins or TRAF-dependent signaling pathways in different human cancers by precision medicine.

## Introduction

The tumor necrosis factor receptor (TNF-R)-associated factor (TRAF 1–7) family of cytoplasmic adaptor proteins regulates the signal transduction pathways of a variety of receptors, including the TNF-R superfamily, Toll-like receptors (TLRs), NOD-like receptors (NLRs), RIG-I-like receptors (RLRs), and cytokine receptors (1–4). TRAF proteins function as both adaptor proteins and E3 ubiquitin ligases to regulate receptor signaling, leading to the activation of canonical and noncanonical nuclear factor-κBs (NF-κB1 and NF-κB2), mitogen-activated protein kinases (MAPKs: ERK1/2, JNK1/2, and p38), or interferon-regulatory factors (IRFs: IRF3, IRF5, and IRF7) (1–4). The TRAF-dependent signaling pathways participate in a diverse array of important cellular processes, including the survival, proliferation, differentiation, activation, and stress responses of different cell types (1–4). Many of these TRAF-dependent signaling pathways have been implicated in cancer pathogenesis.

With the rapid progress made in next-generation deep sequencing technology and the tremendous efforts put forth on whole genome/exome/transcriptome sequencing and copy number variation (CNV) analyses of cancers at the post-genome era, it has become increasingly clear that genetic alterations of TRAF proteins are commonly present in various human cancers. Here we analyze the current evidence of genetic alterations of *TRAF* molecules available from the Cancer Genome Atlas (TCGA) (5) and the Catalog of Somatic Mutations in Cancer (COSMIC) (6) as well as the published literature, including the landscape of genetic alterations and the map of recurrent mutations in *TRAF* molecules in different types of human cancers. Moreover, we summarize the key *in vivo* and *in vitro* evidence that demonstrates the causal roles of genetic alterations of *TRAF* proteins in tumorigenesis within different cell types and organs. Collectively, the information presented in this review identifies *TRAF* proteins and TRAF-dependent signaling pathways as important therapeutic targets in specific human cancers.

## TRAF1

### Landscape of genetic alterations

**Figure 1:**
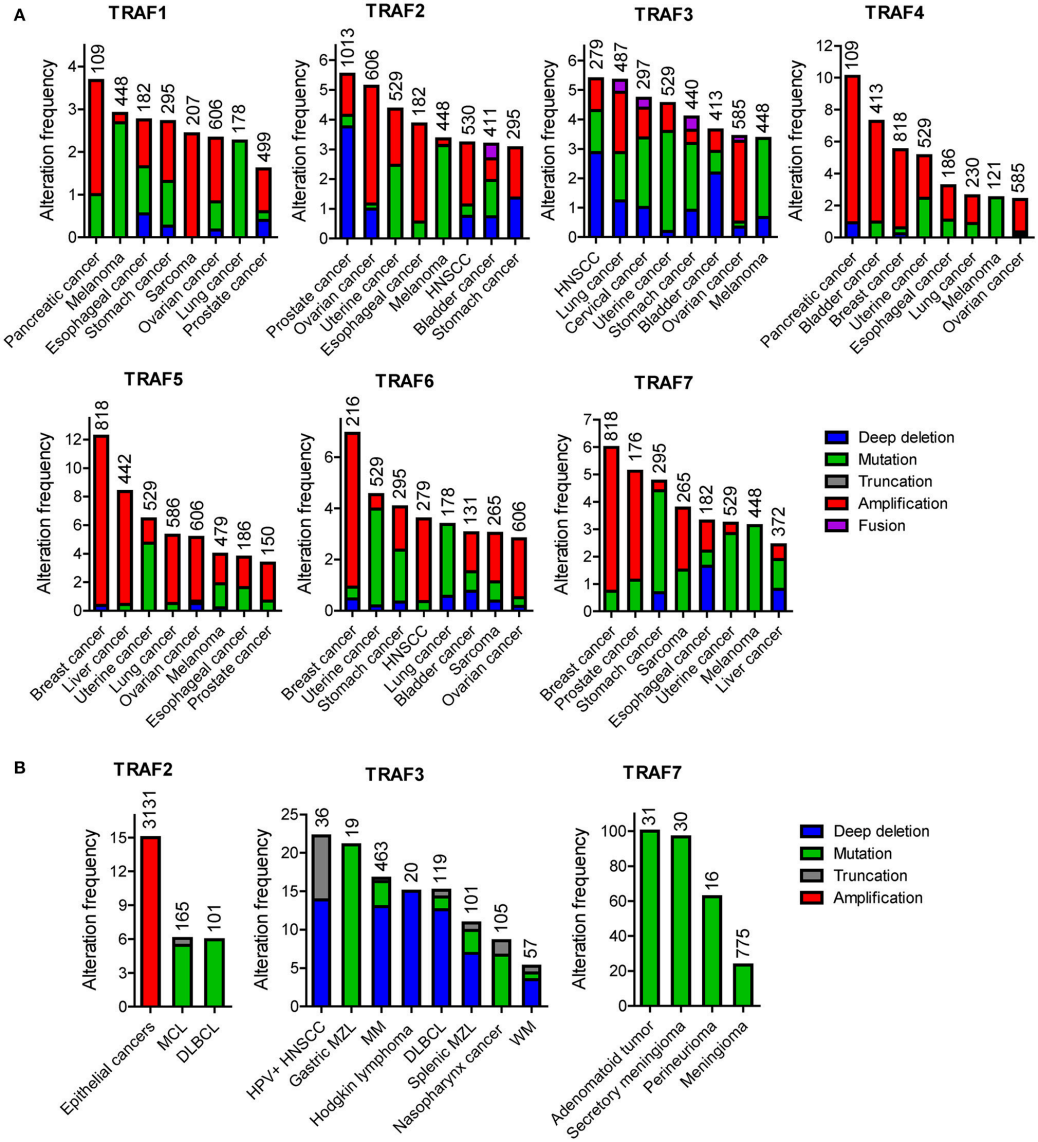
Landscape of genetic alterations of the *TRAF* family in human cancers. **(A)** Representative results retrieved from TCGA. For each *TRAF* gene, eight cancer types that exhibit relatively higher frequency of genetic alterations were selected and datasets with relatively larger sample size (n > 100) are shown. **(B)** Frequent genetic alterations recognized in the published literature. Genetic alterations shown include deep deletion (copy number loss), mutation (missense mutation, frameshift insertion or deletion, and in frame insertion or deletion), truncation (nonsense mutation), amplification (copy number gain), and fusion. The sample size of each dataset is indicated on top of each bar in the graphs.

According to the TCGA and COSMIC datasets of sample size n > 100, the frequency of genetic alterations of *TRAF1* is generally <4% in human cancers (Figure 1A). The eight human cancers with relatively higher genetic alterations of *TRAF1* are pancreatic cancer (3.7%) (7), skin cutaneous melanoma (2.9%) (TCGA, PanCancer Atlas), esophageal cancer (2.8%) (TCGA, PanCancer Atlas), stomach cancer (2.7%) (8), sarcoma (2.4%) (9), ovarian cancer (2.3%) (TCGA, Provisional), lung cancer (2.3%) (10), and prostate cancer (2%) (TCGA, Provisional). The most common genetic alterations of *TRAF1* are gene amplification (copy gain) and mutation. Deep deletion (copy loss) is less common but also detected in several types of human cancers (Figure 1). Truncation is rare for *TRAF1* in human cancers.

### Overview and map of recurrent mutations

**Figure 2:**
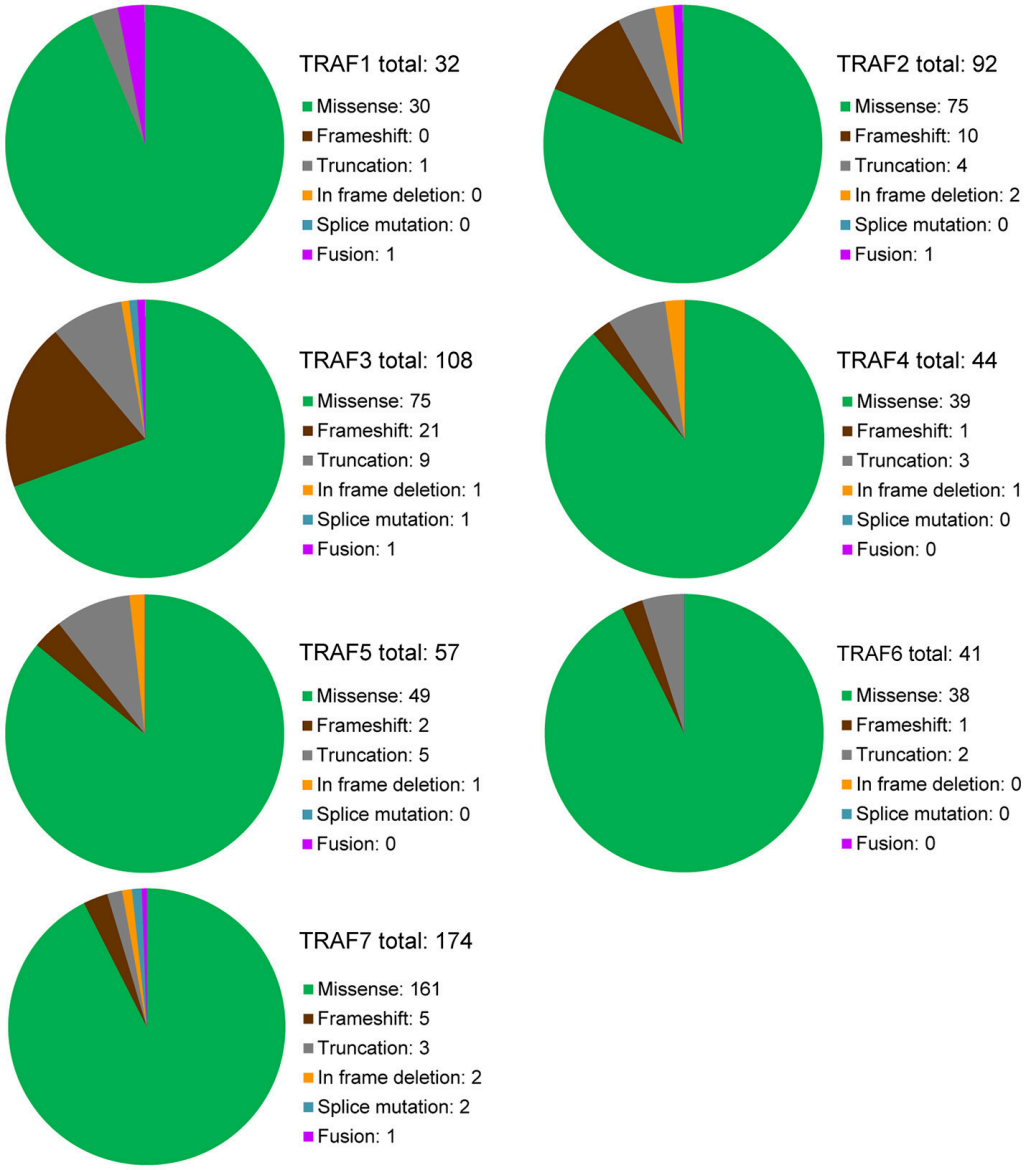
Overview of recurrent mutations of the *TRAF* family in human cancers. Recurrent mutations of the *TRAF* family that are identified in at least 2 cancer patients are summarized in this figure. The composition of recurrent mutation types are shown in a pie graph for each *TRAF* gene. The total count of recurrent mutations and the actual count of each category of recurrent mutation for each *TRAF* gene are indicated in each pie graph.

**Figure 3:**
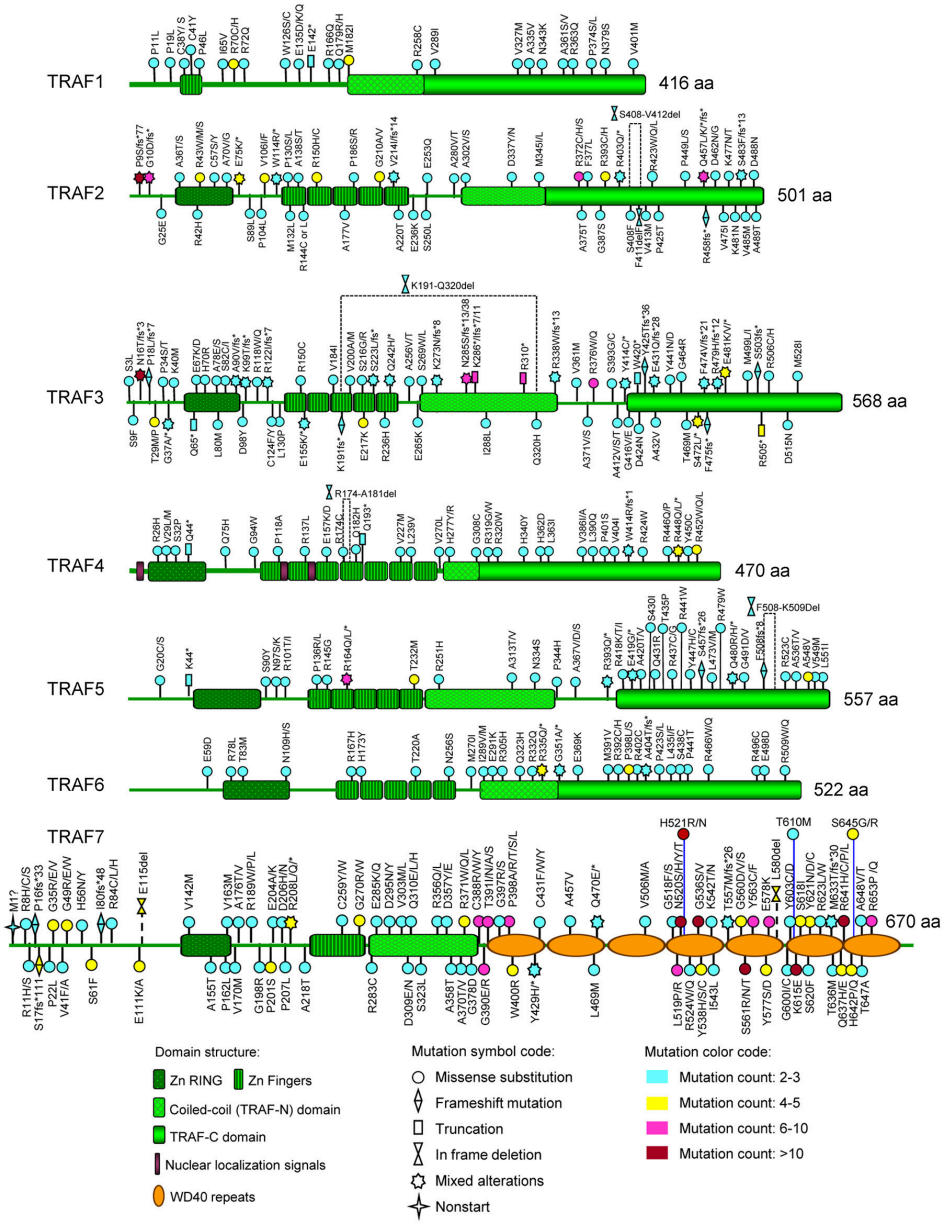
Map of recurrent *TRAF* mutations of human cancers on the TRAF proteins. The distribution of recurrent mutations on the domain structure of each TRAF protein is depicted in this figure. The domain structures of TRAF proteins shown include the zinc RING (Zn RING), zinc fingers (Zn Fingers), coiled-coil (TRAF-N) domain, TRAF-C domain, nuclear localization signals, and WD40 repeats. For each recurrent mutation, the nature of the mutation is indicated by a mutation symbol code and the patient count is indicated by a color code as shown at the bottom legend of the figure. The actual amino acid changes are also given for each recurrent mutation: letter change, missense mutation; *, Nonsense mutation (truncation); fs*, frameshift insertion or deletion; del, in frame deletion.

To date, there are 139 different mutations of the *TRAF1* gene detected in human cancers, comprising 80% (111/139) mutations that alter the protein sequence of *TRAF1* and 20% (28/139) coding silent mutations (Table 1). In the *TRAF* family, *TRAF1* has the lowest count of recurrent mutations. Only 29% (32/111) of the coding-altering mutations of *TRAF1* are recurrent and have been detected in at least two patients with various cancers. Almost all the recurrent mutations of *TRAF1* are missense mutations (94%, 30/32) except one nonsense mutation (truncation) and one fusion (Table 1 and Figure 2). These recurrent mutations occurred across 24 different amino acids that are distributed in all the major domains of the TRAF1 protein (Figure 3). Interestingly, missense mutations of two specific amino acids are detected in more than three patients: R70C or H in the linker between the Zinc finger and the coiled-coil domain, and M182I of the coiled-coil (also known as TRAF-N) domain of the *TRAF1* protein (Figure 3). The R70 mutations are detected in 4 patients with stomach, colon, and colorectal cancers (TCGA) (11–13). M182I is documented in 4 patients with melanoma and chronic lymphocytic leukemia (CLL) (14, 15). The functional significance of R70C/H and M182I mutations of *TRAF1* remains to be determined.

**Table 1:** Summary of the number of different types of mutations of TRAF proteins detected in human cancers.

**Type of mutation**	**TRAF1**		**TRAF2**		**TRAF3**		**TRAF4**		**TRAF5**		**TRAF6**		**TRAF7**	
	**All**	**Recurrent**	**All**	**Recurrent**	**All**	**Recurrent**	**All**	**Recurrent**	**All**	**Recurrent**	**All**	**Recurrent**	**All**	**Recurrent**
**CODING ALTERING**														
Missense	96	30	168	75	166	75	86	39	137	49	132	38	281	161
Frameshift	7	0	13	10	41	21	6	1	8	2	6	1	15	5
Truncation	5	1	9	4	23	9	8	3	9	5	9	2	8	3
In frame deletion	0	0	5	2	2	1	2	1	2	1	1	0	8	2
In frame insertion	0	0	0	0	0	0	0	0	0	0	0	0	2	0
Splice mutation	2	0	5	0	7	1	2	0	4	0	4	0	6	2
Fusion	1	1	5	1	14	1	1	0	0	0	0	0	6	1
Subtotal	111	32	205	92	253	108	105	44	160	57	152	41	326	174
**CODING SILENT**														
Synonymous	24	5	25	3	24	6	18	1	24	5	23	5	39	7
Intronic mutation	4	1	7	0	3	0	0	0	4	2	3	0	11	2
Total	139	38	237	95	280	114	123	45	188	64	178	46	376	183

### Fusion

There is only one fusion of the *TRAF1* gene detected in human cancers, the TRAF1-ALK fusion that has been detected in five patients with anaplastic large cell lymphoma (ALCL) (16–19). All five cases contain the identical in frame fusion of *TRAF1* and *ALK* that generates a chimeric protein linking the N-terminal 1–294 aa of *TRAF1* to the entire intracellular domain of *ALK* (1,058–1,620 aa), including its kinase domain (16–19). Interestingly, expression of the TRAF1-ALK fusion protein leads to constitutive activation of the *ALK* and NF-κB pathways as demonstrated by the elevated levels of phosphorylated *ALK* (pALK) and STAT3 (pSTAT3) as well as nuclear p50 NF-κB1 and p52 NF-κB2 in ALCL cells (18). Similar to wild type (WT) TRAF1, the TRAF1-ALK fusion protein also binds to TRAF2 in co-immunoprecipitation experiments (18), suggesting the involvement of *TRAF2* in the activation of NF-κB pathways. Furthermore, treatment of patient ALCL cells expressing the TRAF1-ALK fusion protein with proteasome inhibitors that decrease NF-κB1/2 or a selective *ALK* inhibitor (CEP28122) results in significant inhibition on lymphoma growth but could not eradicate lymphoma cells (18). Thus, constitutive activation of NF-κB1/2 pathways contributes to the neoplastic phenotype of TRAF1-ALK-expressing ALCL.

### *In vivo* causal oncogenic roles

Gene amplification is the most common *TRAF1* genetic alteration in human cancers. *TRAF1* expression is ubiquitously elevated in skin squamous cell carcinoma (SSCC), non-small cell lung cancer (NSCLC), Hodgkin lymphomas (HLs) and non-Hodgkin lymphomas (NHLs) (20–25). Notably, *TRAF1* protein is consistently elevated in B cell leukemias and lymphomas without evidence of gene amplification (1, 23). In this case, *TRAF1* upregulation might be the result of epigenetic alterations and/or aberrant activation of NF-κB1/2, as *TRAF1* is a direct target gene of NF-κB (23, 26, 27). Interestingly, *TRAF1* expression levels are increased in chronic lymphocytic leukemia (CLL) cells from patients with refractory disease, suggesting a role for *TRAF1* in the progression of this disease and in the development of chemoresistance (23). Furthermore, genetic association studies identify *TRAF1* as a susceptibility gene for risk of CLL (28). Thus, human evidence implicates *TRAF1* as a candidate oncogene. Indeed, *in vivo* evidence obtained from mouse models demonstrates the causal oncogenic roles of *TRAF1* in the skin, lung, T cells, and B cells (Table 2). TRAF1^−/−^ mice exhibit increased skin sensitivity to TNFα-induced necrosis and reduced skin tumor formation induced by DMBA/chronic solar UV radiation (UVR) (20, 29). Mechanistically, TRAF1 enhances the ubiquitination of ERK5 and is required for UVR-induced ERK5 phosphorylation and the expression of AP-1 family members (c-Fos/c-Jun) in keratinocytes and epithelial cells (20). TRAF1^−/−^ mice also show reduced lung tumorigenesis induced by i.p. administration of urethane (30). In this lung cancer model, TRAF1 affects TRAF2-mediated K48-linked ubiquitination and degradation of BRAF, and thereby promotes the survival and proliferation of lung cancer cells (30). Consistent with studies of the TRAF1-ALK fusion protein in ALCL, transgenic mice overexpressing *TRAF1* in T cells exhibit decreased antigen-induced apoptosis of CD8 T cells (35), while TRAF1^−/−^ mice display impaired survival and altered proliferation of T cells in response to the 4-1BB-NF-κB2 and T cell receptor (TCR)-NF-κB1 signaling pathways, respectively (29, 31–34). In line with the evidence of *TRAF1* overexpression in HLs and NHLs, *TRAF1* deficiency inhibits the spontaneous development of small B cell lymphoma in a transgenic mouse model that expresses the human lymphoma-associated NF-κB2 mutant p80HT specifically in lymphocytes (p80HT tg mice) (Table 2) (27). Taken together, these findings identify *TRAF1* as a therapeutic target in skin cancer, lung cancer, and T cell and B cell lymphomas.

**Table 2:** *In vivo* evidence of the causal roles of genetic alterations of the TRAF family in cancer pathogenesis.

**Mouse models**	**Cancer-related phenotype**	**References**
**TRAF1**		
TRAF1^−/−^	Increased skin sensitivity to TNFα-induced necrosis	(29)
	Reduced skin tumors induced by DMBA/solar UVR due to defective UVR-induced	(20)
	ERK5 phosphorylation Reduced lung tumors induced by urethane i.p. administration due to increased	(30)
	TRAF2-mediated ubiquitination and degradation of BRAF	
	Enhanced T cell proliferation in response to TCR-NF-κB1 signaling	(29, 31)
	Impaired CD8 and memory T cell survival in response to 4-1BB-NF-κB2 signaling	(31–33, 34)
TRAF1-tg	Decreased antigen-induced apoptosis of CD8 T lymphocytes	(35)
p80HT tg/TRAF1^−/−^	Reduced development of small lymphocytic lymphoma	(27)
**TRAF2**		
TRAF2^−/−^	Early lethality, reduced TNFα-mediated JNK activation	(36)
	Spontaneous severe colitis and TNFα-dependent apoptosis of colonic epithelial cells	(37)
	Decreased viability of skeletal muscle tissue due to impaired TNFα-induced NF-κB activation in myotubes	(38)
B cell KO: TRAF3flox/flox, CD19-Cre	Prolonged B cell survival, splenomegaly and lymphadenopathy due to constitutive NF-κB2 activation, but defective CD40-induced NF-κB1 activation and proliferation	(39)
B cell tg: Igh-TRAF2DN (ΔN240aa) tg	Lymphadenopathy and splenomegaly due to increased number of B cells	(40, 41)
Igh-TRAF2DN (ΔN240aa)/Bcl-2 tg	Spontaneously development of small lymphocytic lymphoma	(41, 42)
Liver parenchymal cell KO: TRAF2flox/flox, Ripk1flox/flox, Alfp-Cre	Spontaneous development of hepatocellular carcinoma due to extensive hepatocyte apoptosis, caspase 8 hyperactivation and impaired TNFα-induced NF-κB activation	(43)
Induced KO: TRAF2flox/flox, Rosa-creERT2	Rapid lethality that is dependent on Ripk3, TNFR1, DR5 and Fas signaling and increased hepatic necroptosome assembly and necroptosis	(44)
Keratinocyte KO: TRAF2flox/flox, K14-Cre	Psoriatic skin inflammation and epidermal hyperplasia that is partially dependent on TNFα, constitutive NF-κB2 activation and inflammatory cytokine expression	(45)
Myeloid cell KO: TRAF2flox/flox, LysM-Cre	Exacerbated DSS-induced colitis due to increased TLR-induced inflammatory cytokine production caused by elevated c-Rel and IRF5 protein levels in macrophages	(46)
T cell KO: TRAF2flox/flox, Lck-Cre	Decreased NKT cells and CD8 naïve and memory T cells due to impaired IL-15 signaling in NKT cells and defective IL-15-induced proliferation of CD8 T cells	(47)
**TRAF3**		
TRAF3^−/−^	Early lethality, which could be resued by compound loss of p100 NF-κB2 or NIK	(48–50)
	Defective antigen-induced T cell proliferation	(49)
B cell KO: TRAF3flox/flox, CD19-Cre	Expanded B cell compartment, splenomegaly and lymphadenopathy due to prolonged B cell survival caused by constitutive NF-κB2 activation	(539, 51)
	Spontaneous development of splenic marginal zone lymphoma and B1 lymphoma	(52)
	Enhanced signaling by TLR3, TLR4, TLR7, and TLR9 in B cells	(53)
	Accelerated CD40-induced phosphorylation of JNK, p38, and ERK	(54)
B cell Tg: Igh-TRAF3 Tg	Spontaneous plasmacytosis, autoimmunity, inflammation and cancer, particularly squamous cell carcinomas of the tongue and salivary gland tumors	(55)
Myeloid cell KO: TRAF3flox/flox, LysM-Cre	Spontaneous development of histiocytic sarcoma, B lymphoma, liver cancer, or chronic inflammation that often affect multiple organs in aging mice	(56)
	Exacerbated DSS-induced colitis due to increased TLR-induced inflammatory cytokine production caused by elevated c-Rel and IRF5 protein levels in macrophages	(46)
T cell KO: TRAF3flox/flox, CD4-Cre	Impaired T cell proliferation in response to co-engagement of TCR and CD28	(57)
	Increased number of Treg cells due to enhanced IL-2 signaling	(57, 58)
	Impaired IL-15-induced iNKT cell proliferation and survival	(59)
	Reduced number of CD8 central memory T cells due to impaired IL-15 signaling	(60)
**TRAF4**		
TRAF4^−/−^	Defects in embryonic development and neurulation	(61–63)
	Reduced migration of DCs	(64)
	Reduced skin tumors induced by DMBA/TPA due to diminished IL-17A–induced ERK5 activation and epidermal hyperplasia	(65)
	Blunted airway inflammation and Th2 cytokine production in response to IL-25 administration due to defective IL-25R-Act1 signaling	(66)
**TRAF5**		
TRAF5^−/−^	Defective CD40-induced proliferation and surface molecule upregulation in B cells	(67)
	Decreased CD40 plus IL-4-induced Ig production in B cells	(67)
	Impaired CD27-induced survival and proliferation in CD4 and CD8 T cells	(67, 68)
	Defective GITR-induced proliferation, IL-2 production and NF-κB/p38/ERK1/2 activation in CD4 T cells	(69)
	Enhanced OX40-induced Th2 differentiation of CD4 T cells and exacerbated Th2-driven lung inflammation	(70)
	Enhanced IL-6-induced CD4 Th17 differentiation due to increased IL-6-gp130-STAT3 signaling and exaggerated Th17-driven experimental autoimmune encephalomyelitis	(71)
	Exacerbated DSS-induced colitis and increased NF-κB activation in the colon	(72)
CD40LMP1-tg/TRAF5^−/−^	Reduced spleen and LN size compared to CD40LMP1-tg mice, decreased serum IL-6 and autoantibodies, and decreased LMP1-mediated JNK activation in B cells.	(73)
**TRAF6**		
TRAF6^−/−^	Reduced number of immature B cells in the bone marrow	(74)
	Defective differentiation of osteoclasts, DCs, and Treg cells	(74–77)
	Defective IL-1, CD40, LPS and RANK signaling	(74, 75)
	Loss of NF-κB activity in the epithelia and vasculature during development	(78)
	Impaired NGF-p75NTR-induced NF-κB activation and survival in Schwann cells	(79)
	Defective BDNF-p75NTR-induced JNK activation and apoptosis in neurons	(79, 80)
Hematopoietic KO:TRAF6flox/flox, Vav-Cre	Decreased basal IKKβ-NF-κB activation, impaired hematopoietic stem cell self-renewal and loss of hematopoietic stem/progenitor cells (HSPCs)	(81)
B cell KO: TRAF6flox/flox, CD19-Cre	Reduced number of mature B cells in the bone marrow and spleen, defective development of B1 B cells, and defective CD40 and TLR signaling in B cells	(82)
T cell KO: TRAF6flox/flox, CD4-Cre	Multiorgan inflammation and hyperactivation of TCR-PI3K-Akt signaling in CD4 T cells	(83)
	Defects in generating CD8 memory T cells due to impaired AMPK-activation and mitochondrial fatty acid oxidation in response to growth factor withdrawal	(84)
	Increased Th17 differentiation due to increased sensitivity of CD4 T cells to TGFβ-induced Smad2/3 activation and proliferation arrest	(85)
	Impaired OX40-induced Th9 differentiation due to defective OX40-NIK-NF-κB2 signaling	(86)
Intestinal epithelial cell KO: TRAF6flox/flox, Villin-Cre	Exacerbated DSS-induced colitis due to altered gut microbiota, which is independent of TLR signaling in intestinal epithelial cells	(87)
Skeletal muscle KO:TRAF6flox/flox, MCK-Cre	Minimal muscle loss in response to transplanted tumor growth due to defective activation of NF-κB, ubiquitin-proteasome and autophagy-lysosomal systems	(88)
	Improved regeneration of myofibers upon injury due to upregulated Notch signaling but downregulated NF-κB activation and inflammatory cytokine production	(89)
	Reduced starvation-induced skeletal muscle atrophy due to increased phosphorylation of Akt and FoxO3a and decreased AMPK activation	(90)

*Direct evidence in tumorigenesis is highlighted in blue font*.

### Key oncogenic pathways

In addition to the above *TRAF1*-dependent oncogenic pathways (ERK5-AP1, BRAF-ERK, NF-κB1, and NF-κB2) that have been verified in both human cancers and *in vivo* mouse models, several oncogenic pathways involving TRAF1 have been suggested by studies using patient samples, cultured human cancer cells or xenograft models. These include: (1) CD30-TRAF1 in HL and ALCL tumors (22, 91); (2) TNF-R1/2-TRAF1/TRAF2-JNK/NF-κB in cervical and colon cancer cells (92); (3) Wnt/β-catenin-NF-κB-TRAF1/iNOS in colon, breast and liver cancer cells (93, 94); and (4) TWEAK-Fn14-TRAF1 in solid tumors (95–97). Further investigation of these signaling pathways using TRAF1^−/−^ or *TRAF1*-transgenic animal models would provide new insights on the roles and mechanisms of *TRAF1* in cancer pathogenesis.

## TRAF2

### Landscape of genetic alterations

The frequency of genetic alterations of *TRAF2* is generally <6% in human cancers (Figure 1A) based on the TCGA and COSMIC datasets of sample size n > 180. The eight human cancers with relatively higher genetic alterations of *TRAF2* are prostate cancer (5.5%) (98), ovarian cancer (5.1%) (TCGA, Provisional), uterine cancer (4.4%) (TCGA, PanCancer Atlas), esophageal cancer (3.9%) (TCGA, PanCancer Atlas), skin cutaneous melanoma (3.4%) (TCGA, PanCancer Atlas), head and neck squamous cell carcinoma (HNSCC, 3.2%) (TCGA, Provisional), bladder cancer (3.2%) (TCGA, PanCancer Atlas), and stomach cancer (3.1%) (8). Notably, although not cataloged in TCGA, mutations of *TRAF2* are recognized as one of the most frequent somatic mutations in mantle cell lymphoma (MCL, 6.1%, 10/165) (99, 100, 101) and diffuse large B-cell lymphoma (DLBCL, 6%, 6/101) (Figure 1B) (102). In addition, *TRAF2* has been identified as an oncogene that is recurrently amplified and rearranged in 15% of human epithelial cancers (Figure 1B) (103). Thus, the most common genetic alterations of *TRAF2* are deep deletion, gene amplification and mutation (Figure 1). Truncation and fusion of *TRAF2* are relatively rare but also detected in human cancers (Figure 1).

### Overview and map of recurrent mutations

There are 237 different mutations of *TRAF2* detected in human cancers, comprising 86% (205/237) mutations that change the protein sequence of *TRAF2* and 14% (32/237) coding silent mutations (Table 1). Notably, 45% (92/205) of the coding-altering mutations of *TRAF2* are recurrently detected in at least two cancer patients. Recurrent mutations of *TRAF2* are more complex than those of *TRAF1*, including not only missense mutations (82%, 75/92), but also frameshifts (11%, 10/92), truncations (4%, 4/92), in frame deletions (2%, 2/92) and fusion (1%, 1/92) (Table 1 and Figure 2). *TRAF2* recurrent mutations are identified at 52 different amino acids that are almost evenly distributed in all the structural motifs and domains of the TRAF2 protein (Figure 3). Interestingly, four mutation hotspots of *TRAF2* are detected in more than 5 cancer patients, specifically P9, G10, R372, and Q457 (Figure 3). In particular, the frameshift deletion occurred at P9 (P9fs^*^77) is found in 16 patients with colon cancer, colorectal cancer (CRC), uterine cancer, stomach cancer, and sarcoma, and an additional missense mutation at P9 (P9S) is also detected in a CRC patient (TCGA) (12, 104–108). The amino acid right next to P9, G10, also exhibits similar frameshift deletion (G10fs^*^76) or insertion (G10fs^*^70) or missense mutation (G10D) in five patients with colon cancer, CRC, gallbladder cancer, and glioblastoma (TCGA) (105, 106, 109). Missense mutations at R372 (R372C, H or S) of the TRAF-C domain of *TRAF2* are detected in eight patients with HNSCC, melanoma, and prostate, uterine, cervical, stomach, and liver cancers (TCGA; COSMIC) (110–113). Another amino acid of the TRAF-C domain, Q457, shows complex mutations, including a truncation (Q457^*^), a frameshift insertion (Q457fs^*^277), and missense mutations (Q457K or L) in six patients of HNSCC, oral squamous cell carcinoma (OSCC), stomach cancer, melanoma, and breast cancer (TCGA; COSMIC) (8, 114). Frameshift mutations occurring at P9 and G10 are functionally equivalent to deletion of *TRAF2*. Missense mutations at R372 and the complex mutations at Q457 of the TRAF-C domain of *TRAF2* are predicted to result in inactivation of the TRAF2 protein (99–102).

### Fusion

There are five different fusions of the *TRAF2* gene detected in human cancers, including *TRAF2-CCDC183* in breast and bladder cancers, *TRAF2-CACNA1B* in bladder cancer, *TMEM141-TRAF2* in breast cancer (TCGA), *TRAF2-NOTCH1* in ovarian cancer (106) and *NTRK2-TRAF2* in melanoma (115). Among these, only the *TRAF2-CCDC183* fusion is recurrently detected in two patients with breast cancer and bladder cancer (TCGA). Functional contribution of these *TRAF2* fusions to cancer pathogenesis is currently unclear.

### *In vivo* tumor suppressive roles

Inactivating mutations of *TRAF2* are frequently detected in human MCL and DLBCL, resulting in elevated activation of NF-κB1 and NF-κB2 in malignant B cells (99–102). *TRAF2* is also involved in human MALT lymphomagenesis induced by the oncogenic cIAP2-MALT1 fusion protein through the interaction between TRAF2 and the BIR1 domain of cIAP2 portion of the fusion protein, leading to activation of the TRAF2-RIP1-NF-κB pathway (116). Consistent with the human evidence, B cell-specific TRAF2^−/−^ (B-TRAF2^−/−^) mice exhibit expanded B cell compartment in lymphoid organs due to constitutive NF-κB2 activation and survival advantage independent of the B cell survival factor BAFF (Table 2) (117). Similarly in TRAF2DN-tg mice that express a dominant negative form of *TRAF2* specifically in lymphocytes (Igh-TRAF2DN), inhibition of TRAF2 also leads to splenomegaly and lymphadenopathy due to constitutive NF-κB2 activation and increased numbers of B cells (40, 42). Remarkably, TRAF2DN/Bcl-2 double-transgenic mice spontaneously develop small B cell lymphoma progressing to leukemia with many similarities to human CLL (Table 2) (41, 42). Thus, TRAF2 acts as a tumor suppressor in B lymphocytes primarily by inhibiting the NF-κB2 pathway through the well-established cIAP1/2-TRAF2-TRAF3-NIK axis (48, 54, 118).

Genetic alterations of *TRAF2* are detected in 1–2% of human liver cancers, including deletion, mutation and amplification (TCGA, PanCancer Atlas) (119). In human hepatocellular carcinoma (HCC), low expression of *TRAF2* and its interacting partner RIP1 is associated with an unfavorable prognosis (43). In line with human evidence, deletion of both *TRAF2* and RIP1 in liver parenchymal cells (LPC) leads to spontaneous development of hepatocellular carcinoma, which results from extensive hepatocyte apoptosis due to hyperactivation of caspase-8 but impaired NF-κB activation induced by TNFα (Table 2) (43). Interestingly, TRAF2 also suppresses TNFα-induced necroptosis in hepatocytes by constitutively interacting with MLKL, thereby disrupting the TNFα-induced RIPK3-MLKL association and necroptosome formation. Induced *TRAF2* deletion in adult mice results in rapid lethality, in conjunction with increased hepatic necroptosome assembly (Table 2) (44). Therefore, TRAF2 protects hepatocytes from death and tumorigenesis by inhibiting both the TNFα-TNFR1-TRADD-FADD-caspase 8 apoptosis and TNFα-TNFR1-RIPK1-RIPK3-MLKL necroptosis pathways.

Genetic alterations of *TRAF2* are detected in 3–4% of human HNSCC and melanoma (Figure 1A). In cultured HNSCC cell lines, TRAF2 is required for cellular proliferation by acting in the TNFα-TNFR1-TRADD-TRAF2-RIPK1-TAK1-IKK-NF-κB pathway (120). In primary human keratinocytes, exposure to UV light triggers association of TRAF2 with TNF-R1 to induce NF-κB activation and inflammation (121). Keratinocyte-specific TRAF2^−/−^ mice exhibit psoriatic skin inflammation associated with apoptotic death and epidermal hyperplasia, which is dependent on TNFα, constitutive NF-κB2 activation and inflammatory cytokine production (45). Further in support of a role for TRAF2 in skin tumorigenesis, mutations of the TRAF2-deubiquitinating enzyme CYLD are identified in patients with familial cylindromatosis, a condition that results in benign tumors of skin appendages, and CYLD^−/−^ mice are highly susceptible to chemically induced skin tumors (122). Similarly, genetic alterations of *TRAF2* are also identified in 2.7% (12/439) of human colon cancers (TCGA, PanCancer Atlas). In cultured primary human colon cancer cells, TRAF2 mediates the apoptosis by acting in the AMPK-ASK1-TRAF2-JNK-p53 axis in response to chemotherapies (123). Consistent with a potential role of TRAF2 in colon tumorigenesis, germline TRAF2^−/−^ mice spontaneously develop severe colitis, which results from TNFα-TNFR1-mediated apoptosis of TRAF2^−/−^ colonic epithelial cells and altered colonic microbiota (37). Interestingly, myeloid cell-specific ablation of *TRAF2* markedly exacerbates DSS-induced colitis in mice due to enhanced TLR-induced proinflammatory cytokine expression in macrophages (46). This is caused by constitutively elevated levels of the transcription factors c-Rel and IRF5 that are targeted for proteasome-dependent degradation by the cIAP1/2-TRAF2-TRAF3 E3 ubiquitin ligase complex (46). Together, the above evidence consistently supports a suppressive role for TRAF2 in skin and colon tumorigenesis.

It is also noteworthy that genetic alterations of *TRAF2* are detected in 2.6% (7/265) of human sarcomas (TCGA) and TRAF2^−/−^ mice display decreased viability of skeletal muscle tissue because of defective TNFα-induced NF-κB activation in myotubes (Table 2) (38). Additionally, specific deletion of *TRAF2* in T cells results in decreased numbers of CD8 naïve and memory T cells as well as NKT cells, due to impaired IL-15-induced signaling in these cells (Table 2). However, evidence of *TRAF2* genetic alteration in T cell neoplasms is still lacking. Potential causal roles of *TRAF2* dysregulation in muscle or T cell tumorigenesis remain to be elucidated.

### Key signaling pathways in cancer pathogenesis

In addition to the above TRAF2-dependent tumor suppressive pathways verified in both human cancers and *in vivo* mouse models, several important tumor suppressive pathways involving TRAF2 have been suggested by evidence obtained from cultured human cancer cells or xenograft models. These are: (1) IRE1α-TRAF2-ASK1-JNK in the apoptosis of melanoma, lung cancer and OSCC cells induced by chemotherapies or ER stress (124–126); (2) the TRAF2-caspase-2 complex in mediating DNA damage- or chemotherapy-induced apoptosis of breast, cervical and lung cancer cells, in which TRAF2-mediated ubiquitination of caspase-2 stabilizes the caspase-2 dimer complex and enhances its activity to fully commit the cell to apoptosis (127, 128); (3) TRAF2-mediated inhibition of constitutive NF-κB2 activation, cell proliferation, and anchorage-independent growth in pancreatic cancer, and a similar TRAF2-mediatied inhibition of the Eva1-induced NF-κB2-Sox2/CD15/CD49f pathway in the stemness of glioblastoma (129, 130); and (4) TRAF2-mediated K63-linked ubiquitination of MLST8 that disrupts the MLST8-SIN1-mTORC2-Akt pathway in the Kras-driven lung tumorigenesis (131). Together, these data suggest that *TRAF2* is a tumor suppressor in many human cancers.

Interestingly however, increasing evidence indicates that TRAF2 also plays oncogenic roles in epithelial cancers and some other neoplasms. Consistent with the frequent amplification of *TRAF2* detected in human epithelial cancers (Figure 1B) (103), *TRAF2* expression is higher in prostate cancer (133), pancreatic cancer (132), lung cancer (134), stomach cancer (135), colon cancer (136), glioblastoma (137) than in normal tissues. Increased *TRAF2* expression is recognized as a prognostic factor in pancreatic cancer (132), stomach cancer (135), and glioblastoma (137). Importantly, suppression of *TRAF2* in cancer cells harboring a *TRAF2* copy number gain inhibits proliferation, NF-κB activation, anchorage-independent growth, and tumorigenesis (103). Knockdown of *TRAF2* also enhances TRAIL-induced apoptosis in prostate cancer (133) and inhibits the growth but induces radiosensitization of lung cancer and glioblastoma cells (134). Thus, TRAF2 is required for the maintenance of the malignant state in certain cancer cells containing *TRAF2* amplification or overexpression, and TRAF2 protein levels also regulate the sensitivity of cancer cells to chemotherapy and radiotherapy.

A variety of TRAF2-dependent oncogenic pathways have been reported based on studies of patient samples, cultured human cancer cells or xenograft models. Examples include: (1) TRAF2-NEMO-p65-NF-κB1-Bcl2/XIAP/Survivin/TNFα/IL-1/IL-8/HIF-1α in the migration, invasion, metastasis, or drug resistance of breast, stomach and pancreatic cancer cells as well as DLBCL (135, 138–140); (2) EGF-EGFR-TRAF2-RSK2-AP1 in the growth of colon cancer cells (136) and EGFR-TRAF2-RIP1-IKK-NF-κB1 in the resistance to chemotherapy (EGFR inhibitors) in lung cancer cells (141); (3) cIAP1-cIAP2-TRAF2-IKKε-TBK1-IRF3/7/NF-κB1/STAT3 in the tumorigenesis of breast cancer, in which IKKε is amplified in 30% of patients (142, 143); (4) Although TRAF2 is generally considered as a K63-specific E3 ubiquitin ligase (144), a few studies reported TRAF2-mediated K48-linked ubiquitination and degradation of Caspase 8 in the switch of the DR5-Caspase 8 apoptotic pathway to the DR5-Cbl-TRAF2-JNK-AP1-MMP1 invasion/metastasis pathway or the cytoprotective TRAF2-RIPK1-JNK autophagic survival pathway following TRAIL treatment in HNSCC, prostate, lung, stomach, colorectal, and bladder cancer cells (145–148); (5) S100A9-CD147-TRAF2-cdc42 in the metastasis of melanoma (149); (6) TNFα-TRAF2-NF-κB1/AP1-COX2/IL-6/IL-8-PGE2-NOS2 and NOS2-NO-IRE1α-TRAF2-NF-κB1/AP1-COX2/IL-6/IL-8-PGE2 in the growth of breast cancer (150); (7) CD95-TRAF2-NF-κB1/AP1-IL-8/uPA in the invasion of pancreatic cancer (132); and (8) TWEAK-Fn14-TRAF2-SGEF-RhoG-Rac1 in the migration and invasion of glioma (151). Taken together, it is perplexing that both tumor suppressive and oncogenic roles of TRAF2 have been reported in the same type of human cancers. The exact roles of TRAF2 may be dependent on the genetic alteration context and malignant stage of the cancer cells as well as the nature of the environmental cue and treatment regimen.

## TRAF3

### Landscape of genetic alterations

The frequency of genetic alterations of *TRAF3* is generally <6% in human cancers (Figure 1A) according to the TCGA and COSMIC datasets of sample size n > 250. The eight human cancers with relatively higher genetic alterations of *TRAF3* are HNSCC (5.4%) (113), lung cancer (5.3%) (TCGA, PanCancer Atlas), cervical cancer (4.7%) (TCGA, PanCancer Atlas), uterine cancer (4.5%) (TCGA, PanCancer Atlas), stomach cancer (4.1%) (TCGA, PanCancer Atlas), bladder cancer (3.6%) (152), ovarian cancer (3.4%) (TCGA, PanCancer Atlas), and skin cutaneous melanoma (3.4%) (TCGA, PanCancer Atlas). Interestingly however, a subgroup among the 279 cases of HNSCC cataloged in TCGA, the human papilloma virus-positive (HPV+) HNSCC tumors, has much higher frequency (22%, 8/36) of deep deletions and truncations of *TRAF3* than the HPV- HNSCC tumors (Figure 1B) (113). Notably, although not cataloged in TCGA, deletions and mutations of *TRAF3* are recognized as one of the most frequent genetic alterations in a variety of B cell malignancies (153), including gastric marginal zone lymphoma (MZL, 21%) (154), multiple myeloma (MM, 17%) (155, 156), HL (15%) (157), DLBCL (14.3%) (158), splenic MZL (10%) (159), and Waldenstrom's macroglobulinemia (WM, 5.3%) (160) (Figure 1B). Furthermore, somatic mutations of *TRAF3* are also frequently detected in human nasopharyngeal cancer (NPC, 8.6%) (161) (Figure 1B). Together, the most common genetic alteration of *TRAF3* is deep deletion, followed by mutation and then amplification. Truncation and fusion of *TRAF3* are less common but also detected in several different types of human cancers (Figure 1).

### Overview and map of recurrent mutations

There are 280 different mutations of *TRAF3* detected in human cancers, comprising 90% (253/280) mutations that change the protein sequence of TRAF3 and 10% (27/280) coding silent mutations (Table 1). Approximately 43% (108/253) of the coding-altering mutations of *TRAF3* are recurrently detected in at least two cancer patients. Among all the *TRAF* genes, *TRAF3* recurrent mutations exhibit the most complex pattern and include the highest frequencies of frameshift mutations (19%, 21/108) and truncations (8%, 9/108). *TRAF3* recurrent mutations also include 69% (75/108) missense mutations, 1% (1/108) in frame deletion, 1% splice mutation, and 1% fusion (Table 1 and Figure 2). These recurrent mutations occurred at 67 amino acid positions that are distributed in almost the entire length of the TRAF3 protein (Figure 3).

Five mutation hotspots of *TRAF3* are identified in more than 5 cancer patients, specifically N16, N285, K286, R310, and R376 (Figure 3). *TRAF3* mutations at N16 have the highest patient count, including the missense mutation (N16T) identified in 10 patients with HNSCC (COSMIC) and the frameshift deletion (N16fs^*^3) detected in a patient with splenic MZL (162, 163). Mutations at the two consecutive amino acids N285 and K286 of the coiled-coil domain of TRAF3 exhibit the most complex pattern. N285 contains frameshift deletion (N285fs^*^38), frameshift insertion (N285fs^*^13) and missense mutation (N285S) identified in 8 patients with HNSCC, MZL, NPC, CRC, stomach cancer and uterine cancer (TCGA; COSMIC) (12, 107, 161, 164, 165). Similarly, K286 exhibits frameshift deletion (K286fs^*^7 or fs^*^11) and truncation (K286^*^) detected in six patients with B cell malignancies, including MM, CLL and WM (155, 160, 166, 167). A third amino acid of the coiled-coil domain, R310, is consistently targeted by truncation (R310^*^) as detected in 8 patients with DLBCL, MM, HNSCC, cervical cancer and uterine cancer (TCGA) (113, 155, 158, 166, 168). Missense mutations at R376 (R376W or Q) located in the linker between the coiled-coil and TRAF-C domains of TRAF3 are detected in six patients with lung cancer, CRC, SSCC, and melanoma (TCGA; COSMIC) (14, 108, 169). Many of these truncations, frameshifts and missense mutations have been shown to result in inactivation of TRAF3 by disrupting its interaction with NIK, thereby inducing constitutive NF-κB2 activation (155, 156, 158, 159, 170). Thus, most of the recurrent genetic alterations of *TRAF3* identified in human cancers cause complete loss or inactivation of the TRAF3 protein.

### Fusion

There are 14 different fusions of *TRAF3* detected in human cancers, including *TRAF3-WDR20* in stomach and uterine cancers, four fusions of *TRAF3-MYO16, TRAF3-RCOR1, TRAF3-KLC1*, and *EVL-TRAF3* in breast cancer, *TRAF3-SFXN1* in cervical cancer, *UBR5-TRAF3* in HNSCC, two fusions of *TRAF3-ZNF839* and *TRAF3-MARK3* in kidney cancer, two fusions of TRAF3-BMP3 and *SLC22A23-TRAF3* in lung cancer, *TRAF3-IFNL1* in ovarian cancer, *TRAF3-ITPK1* in pheochromocytoma and *TRAF3-SIVA1* in stomach cancer (TCGA). Among the 14 fusions, only the *TRAF3-WDR20* fusion is recurrently detected in two patients with stomach cancer and uterine cancer (TCGA, PanCancer Atlas). However, the functional significance of these TRAF3 fusions is currently unknown.

### *In vivo* causal roles in cancer pathogenesis

Similar to *TRAF2* and also consistent with the frequent deletions and inactivating mutations of TRAF3 identified in human B cell malignancies (Figure 1B), a tumor suppressive role for TRAF3 in B lymphocytes has been demonstrated by *in vivo* evidence obtained from mouse models. As shown for B-TRAF2^−/−^ mice, B cell-specific TRAF3^−/−^ (B-TRAF3^−/−^) mice also exhibit severe peripheral B cell hyperplasia due to prolonged survival of mature B cells independent of BAFF, which results from constitutive NF-κB2 activation (39, 51). These B-TRAF3^−/−^ mice spontaneously develop splenic MZL or B1 lymphoma at high incidence (52). Interestingly, B-TRAF3^−/−^ mice also have doubled number of plasma cells due to enhanced responsiveness to IL-6 (171). Mechanistically, TRAF3 inhibits the IL-6-IL-6R-JAK1-STAT3 survival and differentiation pathway in plasma cells by facilitating the association of PTPN22 with JAK1 (171). Furthermore, the EBV-encoded oncoprotein LMP1 sequesters TRAF3 to produce functional TRAF3 deficiency in human and mouse B lymphoma cells (172, 173). Intriguingly, lymphocyte-specific TRAF3 transgenic mice also develop plasmacytosis, autoimmunity, inflammation, and cancers, which are likely caused by hyper-responsiveness of B cells to antigens and TLR agonists (55). Thus, TRAF3 acts as a tumor suppressor in naïve B cells, but an appropriate and balanced level, neither too high nor too low, of TRAF3 is required to maintain the homeostasis of plasma cells and protect them from tumorigenesis.

Interestingly, specific deletion of TRAF3 from myeloid cells (granulocytes, monocytes, and macrophages) leads to spontaneous development of histiocytic sarcomas derived from TRAF3^−/−^ tissue-resident macrophages in aging mice (56, 174). The pathogenic mechanisms are likely related to the enhanced TLR-induced inflammatory responses observed in TRAF3^−/−^ macrophages through constitutive activation of NF-κB2, c-Rel, and IRF5, as described for TRAF2^−/−^ macrophages (56, 174). Two other mouse models with functional relevance to TRAF3, Dok1^−/−^Dok2^−/−^Dok3^−/−^ mice and humanized TLR7/TLR8 transgenic mice, also spontaneously develop histiocytic sarcomas (175, 176). DOK3, a negative regulator of protein tyrosine kinase (PTK)-mediated signaling, has recently been identified as a TRAF3-interacting protein (177). Similar to TRAF3^−/−^ macrophages, DOK3^−/−^ macrophages are defective in the TLR3-IRF3-IFNβ antiviral pathway (177). TRAF3 is also a transducer of TLR7 and TLR8 signaling through direct interaction with MyD88 (1). Transgenic expression of human TLR7/TLR8 in mice deficient for endogenous TLR7/TLR8 drives inflammation and proliferative histiocytosis, which can be reversed by compound deletion of MyD88 (176). Collectively, the above *in vivo* evidence indicates that TRAF3 is a tumor suppressor in macrophages and that dysregulation of the TLR-MyD88-TRAF3-Dok3 axis in macrophages plays causal roles in the pathogenesis of histiocytic sarcoma. However, because histiocytic sarcoma in humans is a rare malignancy with sparse pathologic and cytogenetic data (178, 179), potential TRAF3 genetic alterations in human histiocytic sarcomas require future investigation.

In addition to the phenotype of histiocytic sarcoma, aging myeloid cell-specific TRAF3^−/−^ (M-TRAF3^−/−^) mice spontaneously develop chronic inflammation and other cancers that often affect multiple organs including the gastrointestinal tract (56). Similar to M-TRAF2^−/−^ mice, young adult M-TRAF3^−/−^ mice exhibit exacerbated DSS-induced colitis with increased levels of inflammatory cytokines produced by TRAF3^−/−^ macrophages in response to TLR signaling (46). Notably, another mouse model with functional relevance to TRAF3, NLRP12^−/−^ mice, is highly susceptible to colitis and colitis-associated colon cancer (180). NLRP12 interacts with both TRAF3 and NIK, and NLRP12^−/−^ cells have constitutively activated NF-κB2 associated with a decreased protein level of TRAF3 (180). Interestingly, both NLRP12^−/−^ hematopoietic and non-hematopoietic cells contribute to inflammation, but the latter dominantly contributes to colon tumorigenesis (180). In line with the *in vivo* data, mutations and deletions of TRAF3 are detected in 2.3% (10/439) of human colon cancers (TCGA, PanCancer Atlas). Furthermore, miR-32-TRAF3-mediated inhibition of the NIK-NF-κB2 pathway protects human colorectal epithelium against colorectal cancer in response to a diet of non-digestible carbohydrates (181). Thus, TRAF3 appears to act in both epithelial cells and myeloid cells to suppress colon tumorigenesis by inhibiting the NF-κB2 and TLR-induced inflammatory pathways.

Although most evidence identifies TRAF3 as a tumor suppressor, studies of the T cell-specific TRAF3^−/−^ (T-TRAF3^−/−^) mouse model suggest an oncogenic role for TRAF3 in T cells. Despite their constitutive NF-κB2 activation, TRAF3^−/−^ T cells exhibit impaired proliferation and activation in response to TCR and CD28 co-stimulation (39, 57). T-TRAF3^−/−^ mice show defects in T cell-mediated immunity and IL-15-induced proliferation and survival of iNKT cells, and also have reduced number of CD8 central memory T cells (Table 2) (57, 59, 60). Consistent with these *in vivo* data, TRAF3 is required for the proliferation of human neoplastic ALCL cells in culture (182). Silencing of TRAF3 in ALCL cells not only results in aberrant activation of the NIK-NF-κB2 pathway, but also affects the continued PI3K-AKT and JAK-STAT signaling (182). Therefore, distinct tumor suppressive and oncogenic roles of TRAF3 in different cellular contexts have been revealed from studies of both human and mouse models.

### Key tumor suppressive pathways

In addition to the above TRAF3-dependent tumor suppressive pathways verified in both human cancers and *in vivo* mouse models, several additional tumor suppressive pathways involving TRAF3 have been suggested by studies of cultured human cancer cells or xenograft models. These include: (1) TRAF3-mediated inhibition of the oncogenic RelB-SMAD4 association that represses TGFβ-SMAD target gene expression to promote the tumorigenesis of lung cancer, in which TRAF3 is targeted by RAS-NDP52-mediated autophagic degradation via the NDP52-TRAF3 interaction (183); (2) LIGHT-LTβR-TRAF3/TRAF5-ROS-ASK1-Caspase3 in the apoptosis of human colon cancer and hepatoma cells (184); (3) membrane-bound CD40L-CD40-TRAF3-p40phox-ROS-ASK1-MKK4-JNK-AP1-caspase 9/3/Bax/Bak in the apoptosis of human bladder and CRC cells but not normal epithelial cells (185–187); and (4) TRAF3-mediated inhibition of the oncogenic RIP2-NF-κB1/NF-κB2/p38-Bcl-xL pathway in the survival and proliferation of glioblastoma cells (188). Taken together, available evidence supports that TRAF3 acts as a tumor suppressor in a variety of cell types, but we cannot rule out that TRAF3 upregulation might also alter normal cell homeostasis in the same or other cell types and therefore contribute to transformation, as it has been observed in B cells and T cells.

## TRAF4

### Landscape of genetic alterations

The frequency of genetic alterations of *TRAF4* is generally <11% in human cancers (Figure 1A) based on the TCGA and COSMIC datasets of sample size n > 100. The eight human cancers with relatively higher genetic alterations of *TRAF4* are pancreatic cancer (10.1%) (7), bladder cancer (7.3%) (152), breast cancer (5.5%) (189), uterine cancer (5.1%) (TCGA, PanCancer Atlas), esophageal cancer (3.2%) (TCGA, Provisional), lung cancer (2.6%) (190), melanoma (2.5%) (191), and ovarian cancer (2.4%) (TCGA, PanCancer Atlas). The most common genetic alteration of *TRAF4* is amplification, followed by mutation (Figure 1). Deep deletion, truncation and fusion of *TRAF4* are relatively rare in human cancers.

### Overview and map of recurrent mutations

There are 123 different mutations of *TRAF4* detected in human cancers, comprising 85% (105/123) mutations that cause changes in the amino acid sequence of *TRAF4* and 15% (18/123) coding silent mutations (Table 1). About 42% (44/105) of the coding-altering mutations of the *TRAF4* gene are recurrent and detected in at least two cancer patients, including mostly missense mutations (89%, 39/44), 3 truncations, 1 frameshift deletion, and 1 in frame deletion (Table 1 and Figure 2). *TRAF4* recurrent mutations occurred at 32 different amino acids that are distributed in the entire length of the *TRAF4* protein but are relatively enriched in the TRAF-C domain (Figure 3). Only two specific amino acids, R448 and R452 located at the C-terminal TRAF-C domain, are mutated in more than 3 patients (Figure 3). For R448, mixed missense mutations (R448Q or L) and a truncation (R448^*^) are identified in 4 patients with prostate cancer, uterine cancer, HNSCC, and OSCC (192–195). For R452, missense mutations (R452W or Q or L) are detected in four patients with uterine, colorectal and lung cancers (10, 108, 193). Further studies are needed to determine whether such missense mutations in the TRAF-C domain result in loss- or gain-of-function of TRAF4.

### Fusion

There is only one fusion of *TRAF4* detected in human cancers, the *TRAF4-FASN* fusion identified in a glioma patient (TCGA), with currently unknown functional significance.

### *In vivo* causal oncogenic roles

Available human evidence indicates that gene amplification is the most common *TRAF4* genetic alteration in cancers and that *TRAF4* expression is ubiquitously elevated in many human cancers (196–204). This suggests that *TRAF4* overexpression may play causal roles in cancer initiation, progression and metastasis. Similar to classical oncogenes (such as c-Myc and K-ras), *TRAF4* is also required for ontogenic processes and TRAF4^−/−^ mice show defects in embryonic development and neurulation (61, 62, 205). Interestingly, TRAF4^−/−^ dendritic cells (DCs) derived from the null mice exhibit reduced *in vivo* and *in vitro* migration (64). Furthermore, recent *in vivo* evidence obtained from mouse models demonstrates the causal oncogenic roles of *TRAF4* in skin tumorigenesis (Table 2) (65). *TRAF4* deficiency substantially diminishes IL-17A-induced ERK5 activation and epidermal hyperplasia in mice. In the DMBA/TPA-induced skin cancer model, TRAF4^−/−^ mice exhibit remarkably reduced tumor incidence and tumor numbers. Mechanistically, *TRAF4* bridges the interaction between Act1 and MEKK3 in response to IL-17A signaling, and therefore is required for the activation of the downstream MEK5-ERK5-Steap4/p63 pathway. The transcription factor p63 directly induces *TRAF4* expression in keratinocytes, promoting positive feedback on TRAF4 in the epidermis and thus sustaining the activation of the TRAF4-ERK5 axis to induce keratinocyte proliferation and skin tumorigenesis (65). These *in vivo* findings are reinforced by the examination of human SSCC samples, which also demonstrates the presence of the IL-17A-Act1-TRAF4-MEKK3-MEK5-ERK5-Steap4/p63 pathway (65). Together, both human and *in vivo* mouse evidence supports an oncogenic role for TRAF4.

### Key oncogenic pathways

In addition to the established IL-17A-TRAF4-ERK5 axis, a variety of potential *TRAF4*-dependent oncogenic pathways have been suggested by studies of patient samples, cultured human cancer cells or their xenografts in immunodeficient mice. These include: (1) TRAF4-Akt/NF-κB-Glut1/HK2/RSK4/Slug in the proliferation and metastasis of lung and breast cancer cells as well as the migration and epithelial-mesenchymal transition (EMT) of hepatocellular carcinoma cells (HCC) (199, 203, 206); (2) TGFβ-TβRI-TRAF4-Smurf1/Smurf2/USP15-SMAD2/TAK1-N-cadherin/Fibronectin/Vimentin/SMA in the migration, EMT, and metastasis of breast cancer cells (200, 207); (3) SRC3-TRAF4-mediated inhibition of the USP7-p53 interaction, leading to the loss of p53 deubiquitination/stabilization and thus the resistance to cytotoxic drugs and stress in breast cancer (208); (4) NGF-TrkA-TRAF4-Akt/p38-IL-6/Integrins/COX2 in the metastasis of prostate cancer cells (204); (5) TNFα-TRAF4/TRAF2-NF-κB1 in the survival and proliferation of breast cancer cells (209); (6) TRAF4-mediated up-regulation and nuclear translocation of β-catenin in the Wnt/β-catenin-cyclin D1/c-myc/Bcl-2/MMPs pathway that promote the growth and migration of OSCC and breast cancer cells (210, 211); (7) TRAF4-mTOR-p70S6K-S6 in the proliferation of breast cancer cells (212); and (8) the TRAF4-phosphoinositide (PIP) interaction at tight junctions that favors breast cancer cell migration (213). It would be interesting to verify these TRAF4-dependent oncogenic pathways using *in vivo* models.

## TRAF5

### Landscape of genetic alterations

The landscape of *TRAF5* genetic alterations is similar to that of *TRAF4*. The frequency of genetic alterations of *TRAF5* is generally <13% in human cancers (Figure 1A) according to the TCGA and COSMIC datasets of sample size n > 140. The eight human cancers with relatively higher genetic alterations of *TRAF5* are breast cancer (12.2%) (189), liver cancer (8.4%) (TCGA, Provisional), uterine cancer (6.4%) (TCGA, PanCancer Atlas), lung cancer (5.3%) (TCGA, Provisional), ovarian cancer (5.1%) (TCGA, Provisional), melanoma (4.0%) (TCGA, Provisional), esophageal cancer (3.8%) (TCGA, Provisional), and prostate cancer (3.3%) (214). As described for *TRAF4*, the most common genetic alteration of *TRAF5* is also amplification, followed by mutation (Figure 1A). Deep deletion, truncation and fusion of *TRAF5* are rare events in human cancers.

### Overview and map of recurrent mutations

There are 188 different mutations of *TRAF5* detected in human cancers, comprising 85% (160/188) mutations that alter the amino acid sequence of *TRAF5* and 15% (28/188) coding silent mutations (Table 1). Approximately 36% (57/160) of the coding-altering mutations of *TRAF5* are recurrent in human cancers. Similar to *TRAF4, TRAF5* recurrent mutations also include mostly missense mutations (85%, 49/57), but also some truncations (9%, 5/57), frameshift deletions (4%, 2/57), and an in frame deletion (2%, 1/57) (Table 1 and Figure 2). These recurrent mutations occurred at 36 different amino acids that are mainly enriched in the TRAF-C domain but also scattered in other regions of the TRAF5 protein (Figure 3). Mutations of three specific amino acids, R164, T232, and A548, are detected in more than three patients (Figure 3). Complex alterations of R164 of the zinc finger motif, including truncation (R164^*^) and missense mutations (R164Q or L), are detected in six patients with uterine, colon and bile duct cancers and DLBCL (TCGA) (12, 193, 215). Another missense mutation of the zinc finger motif, T232M, is detected in four patients with colon, breast, and prostate cancers (TCGA; COSMIC) (98). Missense mutation A548V of the TRAF-C domain is identified in four patients with uterine, cervical, stomach, and breast cancers (TCGA) (107). The functional consequences of these recurrent *TRAF5* mutations await further investigation.

### *In vivo* causal oncogenic roles

Although not cataloged in TCGA, *TRAF5* mutations are detected in 5% (5/101) of human DLBCL (102). *TRAF5* expression is upregulated in human splenic MZL (216). In addition, apoptosis-resistant B cell-acute lymphoblastic leukemia (B-ALL) cells have aberrantly higher protein level of TRAF5 and TRAF6 in response to irradiation than apoptosis-proficient B-ALL cells (217). The above evidence suggests that TRAF5 may be oncogenic in B cells. Consistent with human evidence, B cells of TRAF5^−/−^ mice show defects in CD40-induced proliferation and up-regulation of surface molecules and activation markers as well as CD40 plus IL-4-induced Ig production (Table 2) (67). Using a chimeric CD40-LMP1 transgenic (CD40LMP1-tg) mouse model that mimics the B cell hyperactivation induced by the EBV-encoded oncoprotein LMP1 (218), Kraus et al. demonstrated that TRAF5 is a critical mediator of the *in vivo* functions of LMP1 (73). *TRAF5* deficiency reverses the CD40-LMP1-induced enlargement of the spleen and lymph nodes, decreases the serum levels of IL-6 and autoantibodies that are elevated by CD40-LMP1-tg expression, and also inhibits LMP1-mediated JNK activation in B lymphocytes (Table 2) (73). Together, both human and mouse evidence supports an oncogenic role for TRAF5 in B cells that appears to be required for LMP1-mediated B lymphomagenesis and is likely also involved in spontaneous B lymphomagenesis initiated by genetic alterations.

Additionally, available *in vivo* evidence indicates the importance of TRAF5 in the survival, proliferation and differentiation of different T cell subsets as detailed in Table 2, suggesting that TRAF5 malfunction may contribute to T cell malignancies. However, the evidence of *TRAF5* alterations in human T cell lymphomas/leukemias is still lacking.

### TRAF5-dependent signaling pathways in human cancer cells

In addition to the signaling pathways of B cells and T cells revealed by the *in vivo* studies of TRAF5^−/−^ mice, a number of TRAF5-dependent signaling pathways have been proposed based on the studies of patient samples, cultured human cancer cells or their xenografts in immunodeficient mice. These include: (1) CD30-TRAF2/TRAF5-NIK-IKKα-NF-κB-IL-13 in the survival of Hodgkin-Reed-Sternberg (H-RS) cells of HL (219, 220) and a similar CD30v-TRAF2/TRAF5-NIK-NF-κB pathway in acute myeloid leukemia (AML) and ALL (221); (2) LIGHT-LTβR-TRAF3/TRAF5-ROS-ASK1-Caspase 3 in the apoptosis of human colon cancer and hepatoma cells (184); (3) upregulated TRAF5-NF-κB in the migration and invasion of glioma cells, in which TRAF5 is directly targeted for degradation by the tumor suppressor Numbl (222); (4) TRAF5/TRAF6-NF-κB-Vimentin/TWIST1/SNAIL2/MMP13/IL-11 in the EMT and metastasis of prostate cancer cells, in which TRAF5 is directly targeted for downregulation by the tumor suppressive miR-141-3p (223); and (5) TRAF5-MEK1/2-ERK1/2-Bcl2 in the survival and proliferation of melanoma cells, in which TRAF5 is directly targeted for downregulation by tumor suppressive MiR-26b (224). The above evidence supports the hypothesis that TRAF5 plays oncogenic roles in various human cancer cells primarily by inducing NF-κB activation but also by activating the ERK1/2 pathway.

## TRAF6

### Landscape of genetic alterations

The frequency of genetic alterations of *TRAF6* is generally <7% in human cancers (Figure 1A) based on the TCGA and COSMIC datasets of sample size n > 120. The eight human cancers with relatively higher genetic alterations of *TRAF6* are breast cancer (6.9%) (225), uterine cancer (4.5%) (TCGA, PanCancer Atlas), stomach cancer (4.1%) (8), HNSCC (3.6%) (113), lung cancer (3.4%) (10), bladder cancer (3.1%) (226), sarcoma (3%) (TCGA, Provisional), and ovarian cancer (2.8%) (TCGA, Provisional). Although not listed in TCGA, *TRAF6* amplification is recognized as one of the most frequent genomic alterations in human lung cancer (9.2%, 24/261) (227) and OSCC (10%, 2/20) (228). Consistent with the frequent amplification of *TRAF6* in human cancers, *TRAF6* is overexpressed in many human cancers such as breast cancer, HCC, colon cancer, esophageal cancer, and melanoma (229–233). *TRAF6* overexpression is also identified as a prognostic factor for breast and esophageal cancers (229, 232). Together, the most common genetic alterations of *TRAF6* are mutation and amplification (Figure 1A). Deep deletion of *TRAF6* is less common but also detected in several different types of human cancers. Truncation and fusion of *TRAF6* are rare in human cancers.

### Overview and map of recurrent mutations

There are 178 different mutations of *TRAF6* detected in human cancers, comprising 85% (152/178) mutations that alter the protein sequence of *TRAF6* and 15% (26/178) coding silent mutations (Table 1). Only 27% (41/152) of the coding-altering mutations of *TRAF6* are recurrently detected in at least two cancer patients. Similar to *TRAF1, TRAF6* recurrent mutations also have the simplest composition and are almost exclusively missense mutations (93%, 38/41) except 2 truncations and 1 frameshift insertion (Table 1 and Figure 2). These recurrent mutations occurred at 30 different amino acids that are distributed in all the major domains but are enriched in the coiled-coil and TRAF-C domains of the TRAF6 protein (Figure 3). Mutations of only two specific amino acids, R335 and P398, are detected in more than three patients (Figure 3). A truncation (R335^*^) and missense mutation (R335Q) at R335 within the coiled-coil domain of TRAF6 are detected in five patients with colon and uterine cancers (TCGA) (12, 234). Missense mutations at P398 (P398L or S) of the TRAF-C domain are identified in 4 patients with uterine, lung, and stomach cancers (TCGA) (8, 193, 235). Functional significance of these *TRAF6* recurrent mutations in cancer pathogenesis remains to be elucidated.

### *In vivo* evidence of potential roles for TRAF6 in tumorigenesis

Causal roles of TRAF6 in tumorigenesis have not been directly demonstrated with *TRAF6* knockout or transgenic mice yet. However, available *in vivo* evidence supports potential contributions of TRAF6 dysregulation in tumorigenesis. Consistent with the genetic alterations (mainly amplification and mutation) and frequent overexpression of TRAF6 detected in human epithelial cancers such as breast cancer and uterine cancer (Figure 1A) (229–233), deletion of *TRAF6* in mice results in loss of NF-κB activity in epithelia and vasculature during mouse development (Table 2) (78). Corroborating initial evidence, intestinal epithelial cell-specific TRAF6^−/−^ mice exhibit exacerbated DSS-induced colitis (87). In line with the *in vivo* data, knockdown of TRAF6 or inhibition of TRAF6 E3 ligase activity suppresses the survival, proliferation, migration, and metastasis of many human epithelial cancers, including breast, lung, liver, and colon cancers as well as HNSCC (230–232, 236–240). In the majority of these cases, the TRAF6-NF-κB axis is identified as the main oncogenic pathway, which is constitutively activated by TRAF6 overexpression or hyperactivated by upstream receptors such as TNFα, RANK, and TLR4/3 (236–240).

Similar findings have been obtained in the hematopoietic/lymphoid system. Hematopoietic-specific deletion of *TRAF6* in mice leads to decreased tonic IKKβ-NF-κB activation, impaired hematopoietic stem cell (HSC) self-renewal and loss of hematopoietic stem/progenitor cells (HSPCs) in the bone marrow (BM) (Table 2) (81). Knockdown of TRAF6 in human AML cell lines or patient samples results in rapid apoptosis and impaired malignant HSPC function as well as increased sensitivity to bortezomib (241). In the lymphoid lineage, *TRAF6* mutations have been detected in 2.1% human DLBCL (TCGA) and 2.4% human cutaneous T cell lymphoma (CTCL) (242). TRAF6^−/−^ mice have reduced numbers of immature B cells in the BM and B cells from the null mice show defects in CD40 and LPS-induced proliferation and NF-κB activation (Table 2) (74, 75). B cell-specific TRAF6^−/−^ mice (B-TRAF6^−/−^) mice also exhibit reduced numbers of mature B cells in the BM and spleen as well as defective B1 B cell development (Table 2) (82). Knockdown of TRAF6 or inhibition of the TRAF6-NF-κB axis induces apoptosis and cell cycle arrest in DLBCL, and also inhibits the proliferation and bone resorption of MM (243, 244). Interestingly, T cell-specific TRAF6^−/−^ mice (T-TRAF6^−/−^) mice show multiorgan inflammation due to hyperactivation of the PI3K-Akt pathway in T cells (Table 2) (83). T-TRAF6^−/−^ mice also exhibit increased Th17 differentiation due to enhanced sensitivity of CD4 T cells to TGFβ signaling (85), but have defects in generating CD8 memory T cells caused by defective AMPK activation in activated CD8 T cells (84). In addition, T-TRAF6^−/−^ mice exhibit impaired OX40-induced CD4 Th9 differentiation, which requires TRAF6-mediated activation of the NIK-NF-κB2 pathway in CD4 T cells (Table 2) (86). In support of a role for TRAF6 in T cell tumorigenesis, inhibition of the TRAF6-NF-κB-c-Myc axis through miR-146a/b-mediated downregulation of TRAF6 delays PTEN^−/−^ T cell lymphomagenesis in mice (245). Furthermore, inhibition of the IRAK1/4-TRAF6 axis sensitizes human T cell ALL (T-ALL) to chemotherapies (246). Collectively, the above evidence is consistent with the hypothesis that TRAF6 may serve as an oncoprotein in epithelial cancers and hematopoietic/lymphoid neoplasms mainly through inducing aberrant NF-κB activation.

Interestingly, *in vivo* evidence also indicates the functional importance of TRAF6 in the brain and muscle (Table 2). TRAF6^−/−^ mice show defective neural tube closure and exencephaly (80). Mechanistically, TRAF6 interacts with the p75 neurotrophin receptor (p75NTR), and thus is required for NGF-induced NF-κB activation and survival in Schwann cells as well as BDNF-induced JNK activation and apoptosis in superior cervical ganglia neurons (79). In skeletal muscle, TRAF6 deficiency prevents muscle loss and cancer cachexia in response to transplanted tumor growth, improves regeneration of myofibers upon injury and reduces skeletal muscle atrophy upon starvation through regulating NF-κB activation/ubiquitin-proteasome/autophagy-lysosomal systems, Akt/FoxO3a/AMPK activation and Notch signaling, respectively (88–90). In line with the mouse data, genetic alterations of *TRAF6*, including amplification, mutation and deletion, are detected in 1% of human glioblastoma (247, 248) and 3% of human sarcoma (TCGA) (9). It would be interesting to test potential causal roles of TRAF6 in brain and muscle tumorigenesis in future studies.

### Key signaling pathways in human cancers

In addition to the TRAF6-NF-κB axis that has been verified in both human cancers and *in vivo* mouse models, numerous TRAF6-dependent oncogenic pathways have been reported with studies of patient samples, cultured human cancer cells or their xenografts in mice. Examples are: (1) the TRAF6-p53 crosstalk, in which TRAF6 promotes K63-linked ubiquitination of p53 and limits the tumor suppressive function of p53 in cancer development and resistance to chemotherapy and radiotherapy (249); (2) the Ras-TRAF6-NF-κB axis in the tumorigenesis of lung and pancreatic cancers (227, 250, 251); (3) the TRAF6-Akt axis in the tumorigenesis of glioblastoma, HNSCC, prostate cancer, oral cancer, and CRC (252–255); (4) the EGFR-TRAF6 axis in the growth, migration and metastasis of lung cancer and cutaneous SCC (256, 257); (5) the TRAF6-HIF1α axis in the tumorigenesis, angiogenesis, and metastasis of breast and colon cancers (258, 259); (6) the TGFβ-TβRI/II-TRAF6 axis in the proliferation, migration, and invasion of prostate cancer (260–263); (7) TRAF6-AEP-HSP90α in the invasion and metastasis of breast cancer (229); (8) nutrients-MEKK3-MEK3/6-p38δ-p62-TRAF6-mTORC1 in the growth of prostate cancer (264); (9) pVHL inactivation-CARD9-BCL10-TRAF6-TAK1-MKK4-JNK-AP1-Twist in the EMT of renal cell carcinoma (265); (10) ADAM10-p75NTR ICD-TRAF6 in the metastasis and chemoresistance of HNSCC and breast cancer (266); (11) TRAF6-DNMT1-DNA methylation in chemoresistance of breast cancer (267); and (12) IL-1β-TRAF6-TNFα and TRAF6-Cdc42-F-actin in the invasion of SCC (268). Interestingly, the importance of TRAF6-dependent oncogenic pathways in human cancers is also underscored by the findings that *TRAF6* mRNA is the direct target of tumor suppressive mi-RNAs, including miR-146a (269–274), and miR-141-3p (223). Thus, most evidence indicates that *TRAF6* is an oncogene in human cancers.

Intriguingly, several TRAF6-dependent tumor suppressive pathways have also been described for human cancers in the literature. Examples are: (1) TRAF6-p62-mediated inhibition of the HK2 glycolytic activity and the growth of liver cancer, in which TRAF6 catalyzes the K63-linked ubiquitination of HK2 and targets HK2 for p62-mediated autophagic degradation (275); (2) TRAF6-mediated suppression of the EZH2-H3K27me3 pathway and the progression of prostate cancer, in which TRAF6 mediates K63-linked ubiquitination and degradation of EZH2 (276); and (3) TRAF6-mediated decrease of the H3K4me3 level and thus the tumorigenesis of prostate cancer, in which TRAF6 mediates K63-linked ubiquitination of JARID1B to increase the demethylase activity of JARID1B on H3K4me3 (277). Taken together, both tumor suppressive and oncogenic roles of TRAF6 have been reported in human liver cancer and prostate cancer. As discussed for TRAF2, this phenomenon may be related to the mutational profile and malignant stage of the cancer cells as well as the nature of the environmental cue or treatment regimen.

## TRAF7

### Landscape of genetic alterations

TRAF7 lacks the TRAF homology domain and does not directly interact with any member of the TNFR superfamily, two defining features of the TRAF family (278, 279), and is therefore still controversial to be considered as a genuine member of the TRAF family. The frequency of genetic alterations of TRAF7 is generally <7% in human cancers (Figure 1A) according to the TCGA and COSMIC datasets of sample size n > 150. The eight human cancers with relatively higher genetic alterations of *TRAF7* are breast cancer (6%) (189), prostate cancer (5.1%) (280), stomach cancer (4.8%) (8), sarcoma (3.8%) (TCGA, Provisional), esophageal cancer (3.3%) (TCGA, PanCancer Atlas), uterine cancer (3.2%) (TCGA, PanCancer Atlas), melanoma (3.1%) (TCGA, PanCancer Atlas), and liver cancer (2.4%) (TCGA, PanCancer Atlas). However, it should be noted that although not yet cataloged in TCGA, the rate of *TRAF7* mutation is overwhelmingly high in patients with adenomatoid tumors of the male and female genital tracts (100%, 31/31) (281), secretory meningiomas (97%, 29/30) (282), intraneural perineuriomas (62.5%, 10/16) (283), and meningiomas 23% (182/775) (284) (Figure 1B). In particular, high frequencies (15–26%) of *TRAF7* mutations has been reproducibly detected in multiple studies (284–288), and knowledge of *TRAF7* mutations has contributed significantly to improving the diagnosis, classification, prognosis, and treatment of patients with meningiomas (282, 286, 289–291). Additionally, deletion of *TRAF7* is detected in 67% (18/27) of malignant mesothelioma patients' malignant cells in pleural fluids (292). The most common genetic alteration of *TRAF7* is mutation, followed by amplification and then deep deletion (Figure 1). Truncation and fusion of *TRAF7* are rarely detected in human cancers.

### Overview and map of recurrent mutations

In the *TRAF* family, *TRAF7* has the highest counts of total and recurrent mutations. There are 376 different mutations of *TRAF7* detected in human cancers, including 87% (326/376) coding-altering mutations and 13% (50/376) coding silent mutations (Table 1). Over half (53%, 174/326) of the *TRAF7* coding-altering mutations are recurrently detected in at least two cancer patients. *TRAF7* recurrent mutations are mostly missense mutations (92%, 161/174). Small percentages of other recurrent mutations include 5 frameshifts, 3 truncations, 2 in frame deletions, 2 splice mutations, and 1 fusion (Table 1 and Figure 2). These recurrent mutations occurred at 89 different amino acids covering different regions of the entire length but highly enriched in the last 4 WD40 repeats of the TRAF7 protein (Figure 3). Of particular interest, missense mutations of six specific amino acids located within the C-terminal WD40 repeats, N520, H521, G536, S561, K615, and R641, are identified as mutation hotspots of *TRAF7* detected in more than 15 cancer patients (Figure 3). N520 mutations (N520S, H, Y, or T) are found in 31 patients with meningioma, mesothelioma, sarcoma and colon cancer (12, 106, 282, 284, 285, 293, 294). Mutations of the next amino acid H521 (H521R or N) are identified in 15 patients with adenomatoid tumor, perineurioma, and meningioma (281, 283, 284). G536 mutations (G536S or V) are detected in 16 patients with meningioma, pancreatic cancer, mesothelioma and stomach cancer (106, 282, 284, 285, 293–295). S561 mutations (S561R, N or T) are identified in 19 patients with adenomatoid tumor, perineurioma and meningioma (281, 283, 284). K615E mutations are detected in 15 patients with meningioma and OSCC (284, 296). R641 mutations (R641H, C, P, or L) are detected in 24 patients with uterine, bile duct, colon, stomach and lung cancers and meningioma (TCGA, PanCancer Atlas) (8, 106, 282, 284, 285, 294, 297–299). Although the functional significance of most *TRAF7* mutations is currently unclear, the exceptionally high recurrence and clustering of missense mutations implicate TRAF7 malfunction as a critical pathogenic event in relevant human cancers.

### Fusion

There are six different fusions of *TRAF7* and other genes detected in human cancers, including *TRAF7-LRRC1* in lung cancer, *GFER-TRAF7* in mesothelioma, *CORO7-TRAF7* in glioma, *TRAF7-MAPK8IP3* in bladder cancer, and *TRAF7-RAB26* and *E4F1-TRAF7* in ovarian cancer (TCGA) (106). Among these, only the *TRAF7-LRRC1* fusion is recurrently detected in two patients with lung cancer (TCGA). However, all the *TRAF7* gene fusions have not been verified at the protein level and their functional consequence is unknown.

### *In vivo* evidence of potential roles in cancer pathogenesis

No TRAF7^−/−^ or TRAF7-tg mouse model has been published yet. Importantly however, Tokita et al. recently reported that *de novo* missense mutations in *TRAF7* cause developmental abnormalities and other clinical symptoms in seven unrelated patients, including developmental delay (5/5), congenital heart defects (6/7), limb and digital anomalies (7/7), and dysmorphic facial features (7/7) (300). *TRAF7* mutations identified in this study include a recurrent R655Q mutation found in four patients, and another 3 single mutations each identified in one patient, including K346E, R371G, and T601A (300). Interestingly, R371 recurrent mutations are also detected in human cancers (Figure 3). K346 is a ubiquitination site of TRAF7 (301). Both K346 and R371 are located in the coiled-coil domain of TRAF7 that is important for TRAF7 homodimerization (302, 303). The recurrent R655Q mutation has also been previously identified as a *de novo* event in an autism patient (304). Both T601 and R665 are located in the C-terminal WD40 repeats of TRAF7, which contain most mutation hotspots of *TRAF7* detected in human cancers (Figure 3) and are known to mediate the interaction of TRAF7 with MEKK3 or c-Myb (302, 305). Tokita et al. further revealed that transfection of the R665Q, T601A, or R371G mutants of *TRAF7* into HEK293T cells results in significantly reduced levels of ERK1/2 phosphorylation, both basal and in response to TNFα signaling (300). Consistent with this biochemical evidence, conditional ERK2^−/−^ mice show a phenotype mirroring that observed in the seven patients with *TRAF7* mutations, including craniofacial abnormalities, cardiovascular malformations and limb defects (306). These highly interesting findings warrant further investigation of the *in vivo* functions of *TRAF7* mutations in cancer pathogenesis using animal models.

### TRAF7-mediated signaling pathways

Compared to other TRAF proteins, the signaling mechanisms of TRAF7 are understudied and remain poorly defined (289, 307). In addition to the above TRAF7-ERK1/2 pathway revealed by studying *TRAF7* mutants of patients with developmental defects, the following TRAF7 signaling pathways have been proposed based on *in vitro* studies. (1) Transfection of tumor-derived *TRAF7* mutants (H521R, Y538S, or S561R) but not WT *TRAF7* in 293T cells causes increased phosphorylation of RelA and expression of the NF-κB target gene L1CAM, which is also elevated in adenomatoid tumors (281). (2) Overexpression of *TRAF7* or TNFα induces caspase-dependent apoptosis in HEK293 and HeLa cells via the TRAF7-MEKK3-NF-κB/p38/JNK-AP1/CHOP pathway, in which TRAF7 interacts with MEKK3 and potentiates the kinase activity of MEKK3 (302, 303). (3) TRAF7 mediates TNFα-induced apoptosis in Jurkat and HeLa cells via promoting the K29-, K33-, and K63-linked ubiquitination and lysosomal degradation of c-FLIP, an inhibitor of caspase activation (308). (4) TRAF7 represses TNFα-induced NF-κB activation to enhance apoptosis in HEK293 cells by promoting K29-linked ubiquitination and lysosomal degradation of NEMO and RelA (309). Paradoxically, TRAF7 is identified as an activator of the NEMO-RelA-NF-κB-cyclin D1 pathway in mouse myoblasts and thus a suppressor of myoblast differentiation (310). (5) TRAF7 participates in TLR2-induced production of inflammatory cytokines (TNFα, IL-1β, and IL-8) in A549 and HeLa cells by acting in the TLR2-TRAF6/TRAF7-IKK1/2/NEMO-NF-κB and TLR2-TRAF6/TRAF7-MKK3/6-p38 pathways (311). (6) TRAF7 inhibits the transcriptional activity of the oncoprotein c-Myb in M1 mouse leukemia cells and DND39 human Burkitt's B lymphoma cells by directly interacting with c-Myb and stimulating the sumoylation of c-Myb to sequester c-Myb in the cytoplasm (305). (7) TRAF7 mediates K48-linked ubiquitination of p53 as demonstrated by an *in vitro* ubiquitination assay, which likely induces p53 degradation. Correspondingly, TRAF7 protein is downregulated and p53 protein is upregulated in a panel of breast cancer specimens, and TRAF7 downregulation correlates with poor prognosis in breast cancer (312). In summary, WT TRAF7 appears to be a tumor suppressor that promotes cell apoptosis. *TRAF7* mutations or downregulated protein levels may lead to aberrant NF-κB activation or altered signaling of ERK1/2, p38, JNK, c-FLIP, c-Myb, or p53 to drive tumorigenesis. Further studies are required to clarify the roles and mechanisms of *TRAF7* alterations in cancer pathogenesis.

## Combined genetic alterations of all *TRAFs* in the same human cancer

**Figure 4:**
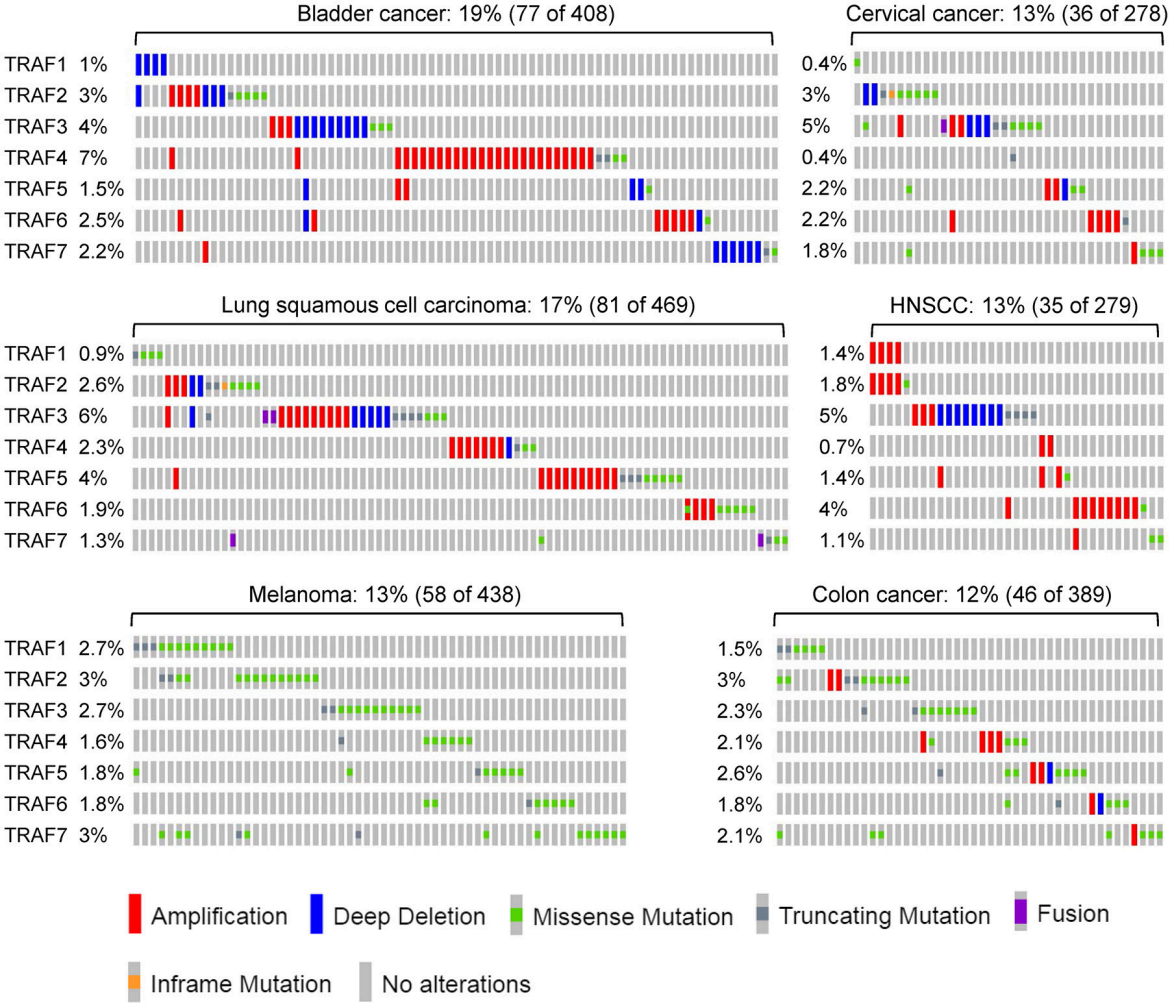
Combined genetic alterations of the *TRAF* family in human cancers. Representative results of the combined genetic alterations of the *TRAF* family in individual human cancers are retrieved from TCGA, specifically bladder cancer, lung cancer, melanoma, cervical cancer, HNSCC, and colon cancer. The sample size and the number of patients containing genetic alterations of *TRAFs* as well as the frequency of each *TRAF* alteration are indicated for each type of cancer in the figure. The nature of *TRAF* genetic alteration identified in each patient is indicated by a mutation symbol as shown at the bottom legend of the figure.

After analyzing the genetic alterations of each *TRAF* gene in human cancers individually, we next examined the combined genetic alterations of all *TRAFs* in the same type of human cancer using the TCGA tool. Although the frequency of the genetic alterations of each *TRAF* is generally low (usually <5%), their combined rate is substantially increased to 10–35% in many types of human cancers (Figure 4) (TCGA). For example, the combined frequency of gene amplification of all seven TRAFs is 35% (709/2015) in breast cancer (313, 314). The combined frequency of genetic alterations of all seven TRAFs is 23% (71/311) in ovarian cancer (TCGA, Provisional), 19% (77/408) in bladder cancer (152), 19% (45/240) in uterine cancer (193), 17% (81/469) in lung cancer (TCGA, PanCancer Atlas), 15% (41/265) in oesophageal cancer (315), 14% (48/353) in liver cancer (TCGA, PanCancer Atlas), 13% (35/279) in HNSCC (113), 13% (36/278) in cervical cancer (TCGA, PanCancer Atlas), 13% (58/438) in melanoma (TCGA, PanCancer Atlas), 12% (46/389) in colon cancer (TCGA, PanCancer Atlas), and 10% (106/1013) in prostate cancer (98). It is interesting that genetic alterations of different *TRAFs* are often mutually exclusive in the same cancer patient and simultaneous genetic alterations of two or three different *TRAFs* in the same cancer patient are generally rare events (Figure 4) except for ovarian cancer (TCGA, Provisional), uterine cancer and melanoma (TCGA, PanCancer Atlas).

**Figure 5:**
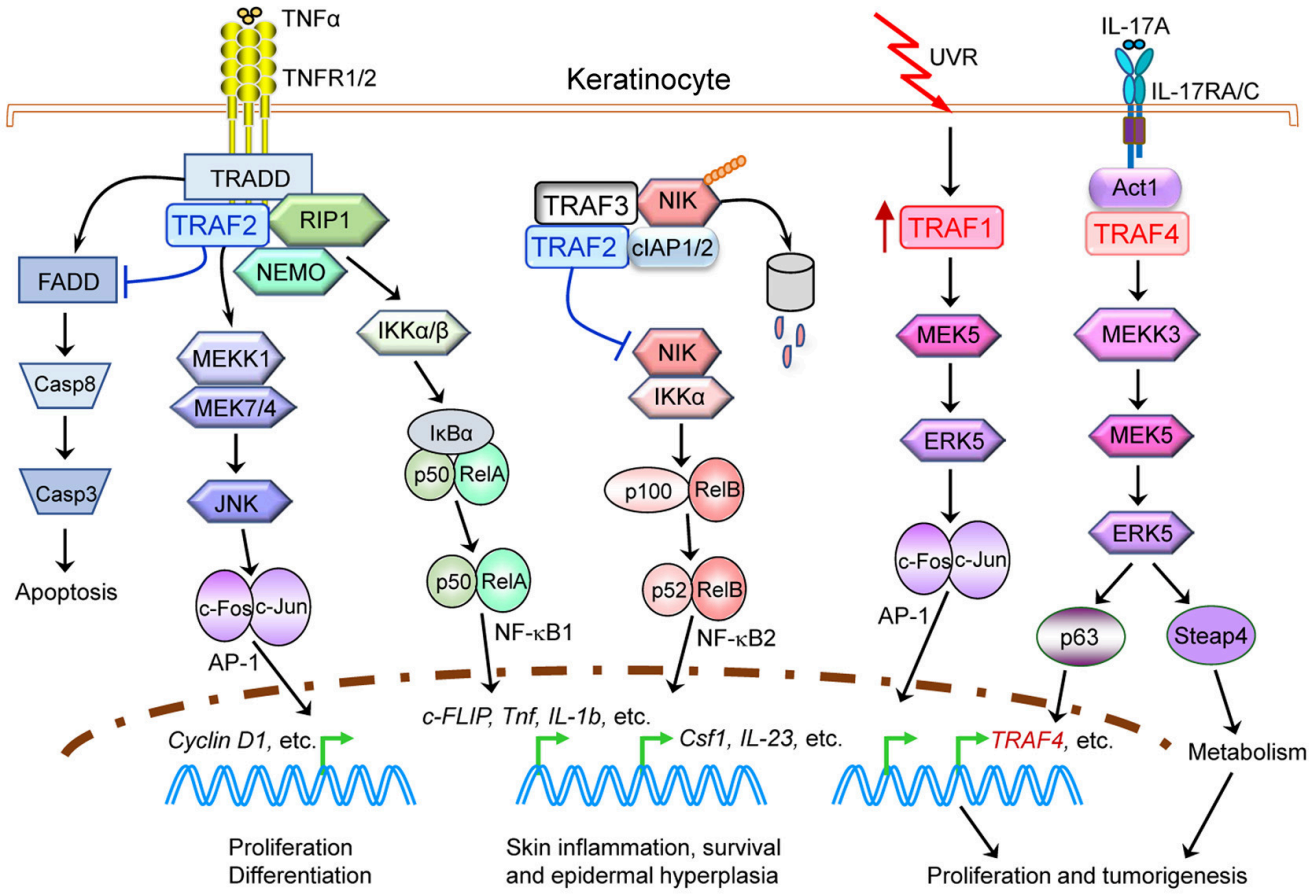
Causal roles and signaling mechanisms of TRAF proteins in skin carcinogenesis. Evidence of both genetic alterations of *TRAFs* in human patients as well as *in vivo* TRAF^−/−^ mouse models indicates that alterations of multiple TRAF proteins, specifically TRAF1, TRAF2, and TRAF4, play causal roles in skin carcinogenesis. Oncogenic TRAF proteins (TRAF1 and TRAF4) are depicted in red, while tumor suppressive TRAF2 proteins are depicted in blue. This figure depicts a simplified model of keratinocyte-intrinsic, TRAF-dependent signaling mechanisms in skin carcinogenesis. Only key TRAF-dependent receptors, TRAF-interacting proteins and downstream kinases and transcription factors that have been verified in both human cancers and *in vivo* mouse models are shown. Keratinocyte-extrinsic, indirect mechanisms of TRAF proteins in skin carcinogenesis are not depicted in the figure, including the known roles of TRAFs in tumor immunity, inflammation and bone resorption and thus their indirect contributions in tumorigenesis and metastasis.

**Figure 6:**
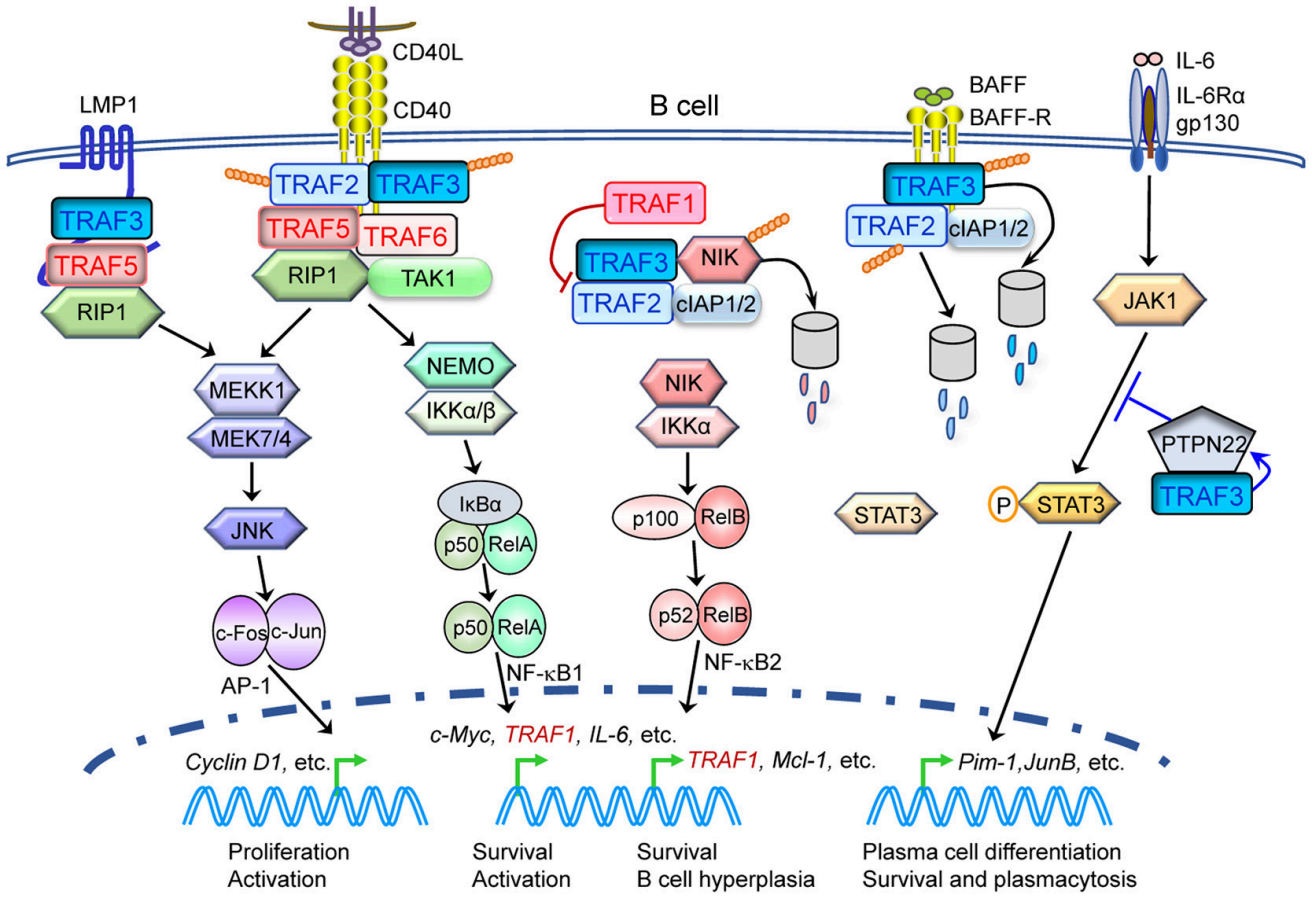
Complex protective and pathogenic roles as well as signaling mechanisms of TRAF proteins in B cell malignancies. Evidence of both genetic alterations of *TRAFs* in human patients as well as *in vivo* TRAF knockout and transgenic mouse models indicates that alterations of multiple TRAF proteins, specifically TRAF1, TRAF2, TRAF3, TRAF5, and TRAF6, play causal roles in the pathogenesis of B cell malignancies, such as B lymphomas and multiple myeloma. Tumor suppressive TRAF proteins (TRAF2 and TRAF3) are depicted in blue, while oncogenic TRAF proteins (TRAF1, TRAF5, and TRAF6) are depicted in red. This figure depicts a simplified model of B cell-intrinsic, TRAF-dependent signaling mechanisms in B cell malignancies. Only key TRAF-dependent receptors, TRAF-interacting proteins and downstream kinases and transcription factors that have been verified in both human cancers and *in vivo* mouse models are shown. Potential contribution of TRAF1, TRAF2, and TRAF6 in LMP1 signaling, TRAF6 in B cell receptor (BCR) signaling and TRAFs in CD40- and LMP1-induced activation of PI-3K-Akt to B cell tumorigenesis are not included in the figure.

We summarize key oncogenic pathways that involve multiple TRAF proteins in skin carcinogenesis as depicted in Figure 5 as well as in B cell malignancies as depicted in Figure 6, both of which have been verified by studies of human cancers and *in vivo* mouse models. However, we believe current understanding only represents “the tip of the iceberg” of oncogenic mechanisms involving TRAF proteins. Given the often mutually exclusive genetic alterations of different TRAFs in the same cancer, it is very likely that all seven TRAFs may have non-overlapping and distinct contributions to different aspects or at different stages of the initiation, progression and metastasis of the same cancer. These unanswered questions represent fascinating areas for future exploration.

## TRAF proteins in pathogen-induced carcinogenesis

The importance of TRAF proteins in cancer pathogenesis is strengthened by mounting evidence that demonstrates their involvement in pathogen-mediated carcinogenesis in certain human cancers. For example, chronic infection with the bacteria *Helicobacter pylori (H. pylori)* is a major cause of gastric cancer. *H. pylori* infection induces TRAF1 overexpression and the expression of the transcription factor Cdx2 in both human gastric epithelial cells and mice, which are mainly driven by NF-κB activation. TRAF1 overexpression plays an antiapoptotic role in *H. pylori*-infected gastric cells (316). Induction of Cdx2 contributes to intestinal metaplasia, a precursor event to gastric cancer. Interestingly, TRAF3 inhibits *H. pylori* infection-induced NF-κB activation and Cdx2 expression, and is required to resist the infection by acting in the NOD1-RIP2-TRAF3 pathway in gastric epithelial cells (317, 318). Furthermore, the oncoprotein *cag* PAI of *H. pylori* activates NF-κB and induces IL-8 secretion through the TRAF2/TRAF6-NIK-IKK pathway in gastric cancer cells (319). Another carcinogenic factor, Tip-α, of *H. pylori* activates NF-κB by inhibiting the expression of miR-3178, which directly targets TRAF3 mRNA for downregulation, in gastric epithelial cells (320). Therefore, *H. pylori* chronic infection-induced gastric tumorigenesis involves activation of TRAF1, TRAF2, and *TRAF6* as well as inhibition of TRAF3 (Table 3).

**Table 3:** Pathogen-encoded proteins that exploit or target TRAFs to induce carcinogenesis in humans.

**Pathogen proteins**	**TRAFs**	**Mechanisms**	**Cancer type**	**References**
**BACTERIAL ONCOPROTEINS**				
Cag PAI of *H. pylori*	TRAF1, 2, 6	Utilizes TRAF1, 2, and 6 to induce NF-κB activation and IL-8 secretion	Gastric cancer	(316, 319)
Tip-α of *H. pylori*	TRAF3	Induces TRAF3 protein and NF-κB activation by inhibiting miR-3178 expression, which targets TRAF3	Gastric cancer	(320)
**VIRAL ONCOPROTEINS**				
LMP1 of EBV	TRAF1, 2, 3, 5, 6	Sequesters cellular TRAF3, and usurps TRAF1, 2, 3, 5, and 6 to mimic constitutively activated CD40 signaling, induces NF-κB1 and NF-κB2 activation, and induces	B lymphomas	(1, 172, 173, 321)
		EGFR expression	Nasopharyngeal carcinoma	(322–324)
	TRAF5, 6	Recruits TRAF5 and 6 to activate p38 and suppress the replication of EBV, maintaining the lalent state of EBV	Burkitt's lymphoma	(325)
v-FLIP of KSHV	TRAF2, 3	Recruits TRAF2 and 3 to activate NF-κB and JNK, and to induce cell survival	Primary effusion lymphoma	(326)
pUL48 of HCMV	TRAF3, 6	Deubiquitinates TRAF3 and 6 to inhibit type I IFN production, enhances cellular metabolic activity and upregulates anti-apoptotic proteins	Breast cancer, glioma	(327)
E6 protein of HPV	TRAF3	Inhibits p53 and RB expression, but E6 protein levels are inhibited by TRAF3	HNSCC	(328)
Core protein of HCV	TRAF2, 5, 6	Interacts with TRAF2, 5, and 6 to activate NF-κB and induce inflammation	Hepatocellular carcinoma	(329, 330)
Tax of HTLV-1	TRAF3, 6	Interacts with TRAF3 and 6 to induce TBK1-IKKε activation, type I IFN production and Mcl-1 stabilization	T cell leukemia	(331, 332)
**VIRAL TUMOR SUPPRESSORS**				
E2 protein of HPV	TRAF5, 6	Interacts with TRAF5 and 6, promotes K63-linked ubiquitination of TRAF5, and potentiates TNFα-induced NF-κB activation by activating TRAF5	Cervical cancer, HNSCC	(333, 334)

A variety of viral infections have also been linked to cancer development. DNA viruses, such as Epstein-Barr virus (EBV), Kaposi's sarcoma-associated herpesvirus (KSHV), human cytomegalovirus (HCMV) and human papilloma virus (HPV), cause NPC, B lymphomas, breast cancer, glioma, cervical cancer, and HNSCC in the host (335, 336). RNA viruses, such as hepatitis C virus (HCV) and human T-cell leukemia virus type 1 (HTLV-1), may lead to HCC and T cell leukemia, respectively, in an infected individual (335, 336). Notably, oncogenic proteins of these viruses exploit or target one or multiple TRAF proteins for their signal transduction. These include the EBV-encoded oncoprotein LMP1, v-FLIP of KSHV, pUL48 of HCMV, E2 and E6 of HPV, Core protein of HCV and Tax protein of HTLV-1. In particular, consistent with the high frequency of TRAF3 deletions and mutations in HPV+ HNSCC, overexpression of TRAF3 inhibits the growth, migration and chemoresistance of HPV+ HNSCC by decreasing HPV E6 oncoprotein and increasing p53 and RB tumor suppressors (328). We briefly summarize the TRAF-dependent signaling mechanisms of pathogen-encoded proteins that contribute to human carcinogenesis as detailed in Table 3.

## Indirect mechanisms of TRAFs in human cancers

Although beyond the scope of this review, we would like to point out that as critical regulators of adaptive immunity, innate immunity, and inflammation (1–4), TRAF proteins may indirectly contribute to the development, progression, and metastasis of various cancers by affecting tumor surveillance, tumor immunity, chronic inflammation and the tumor microenvironment. For example, disorders of innate antibacterial response are of fundamental importance in the development of gastrointestinal cancers, including pancreatic cancer, and increased expression of TRAF6, TLR4, and NOD1 are detected in peripheral blood leukocytes of pancreatic cancer patients (337). Specific deletion of TRAF3 from myeloid cells leads to development of B lymphomas and liver cancer in mice (56, 174). Similarly, lymphocyte-specific TRAF3 transgenic mice develop autoimmunity, inflammation and cancers (such as squamous cell carcinomas of the tongue, salivary gland tumors, and hepatoma) (55). TRAF2 regulates inflammatory cytokine production in tumor-associated macrophages, which facilitates tumor growth (46). TRAF4 promotes lung cancer aggressiveness by modulating the tumor microenvironment in normal fibroblasts via the TRAF4-NOX2/NOX4/p47-phox-ROS pathway (338). Importantly, TRAFs are also recognized as potential targets or modulators of cancer immunotherapy. For example, the immune adjuvants dsRNA such as Sendai Virus, poly-I:C, and rintatolimod all activate the TLR3-TRAF3-IRF3 axis to promote CD8 cytotoxic T lymphocytes chemotaxis to the tumor microenvironment in cancer immunotherapy (339). Anti-GITR immunotherapy-induced tumor-specific Th9 cells, which are highly effective in eradicating advanced tumors *in vivo*, display a unique hyperproliferative feature driven by the Pu.1-TRAF6-NF-κB axis (340, 341). Furthermore, TRAF3 and TRAF6 are crucial for osteoclast differentiation, and therefore can regulate bone metastasis of various cancers (4, 342). We shall witness rapid advancement in these exciting areas in the coming years.

## Conclusions

In this article, we have analyzed the current evidence of genetic alterations of the *TRAF* family in human cancers. The results revealed that genetic alterations of all seven *TRAF* genes are present in various human cancers and that recurrent mutations of each *TRAF* gene have been detected in cancer patients. In particular, loss-of-function genetic alterations of *TRAF2* and *TRAF3* are frequently detected in B cell malignancies, and the rates of missense mutations of *TRAF7* are overwhelmingly high in adenomatoid tumors, secretory meningiomas and perineuriomas. Gain-of-function alterations (gene amplification and overexpression) are common for *TRAF1, TRAF4, TRAF5*, and *TRAF6* in human cancers, and are also identified for *TRAF2* in epithelial cancers. Corroborating human evidence, direct causal roles of *TRAF* genetic alterations (except *TRAF7*) in tumorigenesis have been demonstrated *in vivo* with genetically engineered mouse models that have each *TRAF* gene deleted or overexpressed in specific cell types. Importantly, however, the functional significance of most *TRAF* point mutations identified in human cancers remains to be assessed in future studies. A number of interesting TRAF-dependent oncogenic and tumor suppressive pathways have been elucidated from both *in vivo* and *in vitro* studies, although current understanding is still far from complete and further investigation is required. The significance of TRAFs in cancer pathogenesis is reinforced by the evidence that TRAF proteins also participate in pathogen-induced carcinogenesis, including bacteria and viruses. Furthermore, emerging evidence indicates that TRAF proteins can indirectly contribute to tumorigenesis and metastasis by affecting tumor immunity, chronic inflammation, bone resorption, and the tumor microenvironment. In conclusion, the information presented in this article provides a rationale for the development of novel immunotherapies and other strategies to manipulate TRAF proteins or TRAF-dependent signaling pathways in human cancers by precision medicine, which represents the next primary challenge in the field.

## Author contributions

PX and SZ have taken the leading roles in designing and writing this manuscript, and all co-authors (JJ, SG, AL, HS, and JF) have also made significant contributions to writing this manuscript.

### Conflict of interest statement

The authors declare that the research was conducted in the absence of any commercial or financial relationships that could be construed as a potential conflict of interest.
